# A hemicyanine-based dual-responsive fluorescent sensor for the detection of lithium and cyanide ions: application in living cells

**DOI:** 10.1007/s00216-025-05852-w

**Published:** 2025-04-03

**Authors:** Ziya Aydin, Mukaddes Keskinates, Esra Armagan, Bahar Yilmaz Altinok, Mevlut Bayrakci

**Affiliations:** 1 Vocational School of Technical Sciences, Karamanoglu Mehmetbey University, 70100 Karaman, Turkey; 2 Department of Environmental Protection Technologies, Kazım Karabekir Vocational School, Karamanoglu Mehmetbey University, 70100 Karaman, Turkey; 3 Department of Pharmacy Services, Ermenek Uysal and Hasan Kalan Health Services Vocational School, Karamanoglu Mehmetbey University, 70400 Karaman, Turkey; 4 Department of Bioengineering, Faculty of Engineering, Karamanoglu Mehmetbey University, 70200 Karaman, Turkey

**Keywords:** Bio-imaging, Chemosensor, Cyanide, Dual-response, Fluorescence, Lithium ions

## Abstract

A hemicyanine-based colorimetric and fluorometric sensor, 2-(2-(2,3,5,6,8,9-hexahydrobenzo[b][1,4,7,10]tetraoxacyclododecin-12-yl)vinyl)-3,3-dimethyl-1-propyl-3H-indol-1-ium iodide (MH-5), was developed and synthesized to detect Li^+^ and CN^−^ ions in DMSO-PBS buffer solution (10 mM, pH 7.25, v/v 1:9). MH-5 displayed a rapid and highly selective colorimetric response to both Li^+^ and CN^−^, indicated by a distinct color change from pink to pale pink in the presence of Li^+^ and to colorless upon CN^−^ detection, without interference from other cations or anions. The interaction mechanisms of MH-5 with Li^+^ and CN^−^ ions were investigated using various analytical techniques, including ^1^H NMR, ESI–MS, FT-IR spectroscopy, and Job’s plot analysis. These studies suggest that CN^−^ is detected through nucleophilic addition to the indolium moiety of MH-5, while Li^+^ detection occurs via coordination with oxygen atoms in the crown ether structure. The fluorescence-based detection limits for Li^+^ and CN^−^ were determined to be 0.150 µM and 0.154 µM, respectively. Additionally, MH-5 was evaluated in living cells, demonstrating effective cell penetration and reliable detection of Li^+^ and CN^−^ ions for potential bio-imaging applications.

**Graphical Abstract:**

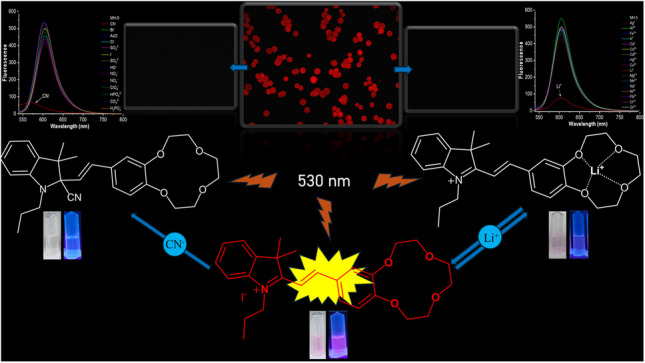

**Supplementary Information:**

The online version contains supplementary material available at 10.1007/s00216-025-05852-w.

## Introduction

Detection of cations and anions has been an important part of modern research due to its widespread applications in biological, chemical, and environmental analysis. Among anions, cyanide (CN^−^) is highly toxic to living organisms due to its strong affinity for binding to the heme group, which disrupts the function of cytochrome c oxidase, inhibiting the process of cellular respiration [1, 2]. This disruption leads to symptoms such as vomiting, seizures, loss of consciousness, and ultimately death. The lethal dose of CN^−^ for humans can range from 0.5 to 3.5 mg per kg of body weight [3]. According to the World Health Organization (WHO), the maximum allowable cyanide concentration in drinking water is 1.9 µM [4]. Despite its toxicity, cyanide is widely used in industrial applications, producing high cyanide content in materials such as paper, textiles, and plastics. Furthermore, some biological processes in bacteria and fungi release cyanide [5, 6]. However, the accidental release of cyanide poses significant environmental and health risks. Therefore, developing efficient, cost-effective methods for cyanide detection is critical.

Among cations, lithium (Li^+^) is one of the most biologically important alkali cations, and lithium-containing drug preparations are routinely used in medical and clinical applications for the treatment of bipolar disorders and manic-depressive psychosis [7–9]; also, there is interest in lithium salts for the treatment of amyotrophic lateral sclerosis (ALS) and Alzheimer’s disease [10]. Lithium plays a vital role in energy storage technologies, particularly in batteries, and its demand is experiencing significant growth [11, 12]. Although the extensive use of lithium has contributed to reducing environmental pollution associated with conventional energy sources, it also poses serious risks. For instance, lithium present in discarded cathode materials can adversely affect human health by leaching into the soil and accumulating in the food chain, leading to potential kidney damage and nephrotic syndrome [13]. Consequently, there is an urgent need to develop and selective methods for the detecting Li^+^ cations.

Over the past few decades, a range of analytical techniques, such as atomic absorption spectroscopy [14, 15], voltammetry [16, 17], and electrochemical detection [4, 18], have been employed for the detection of Li^+^ and CN^−^ ions. However, fluorimetric techniques have garnered significant attention due to their simplicity, high sensitivity, and selectivity compared to these traditional methods [19–24]. Consequently, developing a chemosensor capable of providing a reliable and effective response to Li^+^ and CN^−^ has become a topic of increasing interest in recent studies.

Due to the dedicated efforts of researchers, numerous effective colorimetric and fluorescent sensors have been developed for the selective detection of CN^−^, primarily utilizing mechanisms such as designs involving nanoparticles [25], nucleophilic addition [26], and metal complex ensemble displacement [27, 28]. Among these, sensors based on nucleophilic reactions with functional groups like pyridinium rings [29, 30], dicyano-vinyl [31, 32], and indolium groups [33–37] have demonstrated notable selectivity, attributed to CN^−^, high nucleophilicity. Recently, indolium has shown particular promise as a reactive group for CN^−^ detection due to its positive charge, which creates a strong electrostatic attraction with CN^−^.

In recent literature, various sensors for Li^+^ ions have been developed, typically utilizing chelation-induced photophysical changes, such as photoinduced electron transfer (PET) [38], intramolecular charge transfer (ICT) [39], and Förster resonance energy transfer (FRET) [40]. To construct optical sensors, chemists typically design a system comprising two main components: an ionophore that selectively binds lithium and a chromophore or fluorophore responsible for optical changes [41, 42]. Numerous ligands capable of forming lithium chelates have been explored, including porphyrins [43, 44], phenanthrolines [45], and calixarenes [46, 47], but crown ethers and their derivatives [39, 48–51] are especially known for their strong affinity for lithium. Chromo- and fluorophores used in these systems are often small molecular dyes such as BODIPY [50], coumarins [52], and naphthalimides [38]. When functionalized with crown ethers, these probes generally exhibit complex molecular architectures that require multiple synthetic steps to prepare; however, they offer high selectivity for Li^+^.

With this background and our interest in dual-responsive fluorescent sensors, we decided to study on a turn-off fluorescent, MH-5, synthesized by combining2,3,3-trimethyl-1-propyl-3H-indol-1-ium iodide (the indole moiety) and 2,3,5,6,8,9-hexahydrobenzo[b][1, 4, 7, 10] tetraoxacyclododecine-12-carbaldehyde (the crown ether moiety), for the detection of Li^+^ and CN^−^. The optical and colorimetric properties of the sensor were examined using naked eye observation, fluorescence, and UV–Vis spectrophotometry in DMSO-PBS buffer (10 mM, pH = 7.25, v/v, 1:9). The sensor demonstrated rapid and highly selective detection of Li^+^ and CN^−^ ions among the tested cations and anions. Spectrophotometric methods were employed to analyze the interactions between MH-5 and Li^+^/CN^−^, with ^13^C NMR titration experiments used to clarify the sensing mechanism for CN^−^ and FT-IR experiments for Li^+^. The sensor was further tested for bio-imaging of Li^+^ and CN^−^ in living cells for practical applications.

## Experimental

### Materials and instruments

All solvents and reagents were of analytical reagent grade and were used without further purification. 2,3,3-Trimethyl-3H-indole, 1-iodopropane, 3,4-dihydroxybenzaldehyde, p-Toluene sulfonyl chloride, and triethylene glycol were purchased from Sigma-Aldrich. The metal salts and the anion salts were commercially obtained from Acros Organics or Sigma-Aldrich.

^13^C NMR and ^1^H NMR spectra were acquired using an Agilent Premium Compact spectrometer operating at 600 MHz at a temperature of 298 K. Infrared (IR) spectra were recorded using a Bruker Vertex FT-IR spectrometer with ATR (Attenuated Total Reflectance) capabilities. UV–Vis absorption and fluorescence emission spectra were measured with a Shimadzu UV-1800 spectrophotometer and a Hitachi F-7100 spectrophotometer, respectively, both at 298 K. For cellular imaging experiments, a Leica™ DM 3000 fluorescence microscope was employed to capture cell images.

### Synthesis of 2-(2-(2,3,5,6,8,9-hexahydrobenzo[b][1,4,7,10]tetraoxacyclododecin-12-yl)vinyl)−3,3-dimethyl-1-propyl-3H-indol-1-ium iodide (MH-5)

2,3,3-Trimethyl-1-propyl-3H-indol-1-ium iodide and 2,3,5,6,8,9-hexahydrobenzo[b][1, 4, 7, 10]tetraoxacyclododecine-12-carbaldehyde was synthesized as reported in the literature [53, 54] (also see Electronic Supplementary Material Scheme S1).

2,3,3-Trimethyl-1-propyl-3H-indol-1-ium iodide (**C1**) (0.330 g, 1 mmol) and 2,3,5,6,8,9-hexahydrobenzo[b][1, 4, 7, 10]tetraoxacyclododecine-12-carbaldehyde (**C2**) (0.250 g, 1 mmol) were dissolved in 5.0 mL of ethanol. The mixture was stirred at 100 °C for 1 h. When the substances were completely dissolved, 20 µL (2 drops) of piperidine was added. The mixture was refluxed at 100 °C for 12 h (checked by TLC). After cooling to room temperature, it was kept in the refrigerator at − 20 °C for 1 day. The resulting precipitate was collected by filtration. It was washed with cold ethanol and dried in a vacuum oven. 0.230 g of dark purple MH-5 was obtained (41%). ^1^H NMR (600 MHz, DMSO) δ 7.47–7.30 (5H), 7.05 (m, 1H), 6.85 (dd, *J* = 17.1, 4.7 Hz, 2H), 6.06 (s, 1H), 4.83 (d, *J* = 3.3 Hz, 2H), 3.68–3.54 (m, 12H), 2.10 (dd, *J* = 15.1, 8.3 Hz, 2H), 2.02 (s, 6H), 1.12 (t, *J* = 10.8 Hz, 3H). ^13^C NMR (DMSO-d6, 150 MHz), δ (ppm): 180.0, 161.8, 154.8, 153.4,143.2, 141.4, 129.0, 128.0, 123.2, 122.8, 114.0, 112.9, 105.2, 70.7, 70.1, 68.3, 52.7, 44.1, 26.9, 22.1, 11.0. MALDI-Tof: C_27_H_34_NO_4_^+^ ([M]^+^ without I^−^) found 436.466, calculated 436.245.

### General procedure for detecting spectra

Stock solution of MH-5 was prepared at a concentration of 1.0 mM in dimethyl sulfoxide (DMSO). This solution was further diluted to achieve a final sensor concentration of 1 μM using DMSO-PBS buffer (10 mM, pH = 7.25, v/v, 1:9) solvent system for spectroscopic analysis. Stock solutions of metal ions (10 mM) were prepared using various salts, including AlCl_3_·6H_2_O, AgNO_3_, CaCl_2_, CoCl_2_·6H_2_O, 3CdSO_4_·8H_2_O, CuCl_2_·2H_2_O, CrCl_3_·6H_2_O, HgCl_2_, FeCl_3_·6H_2_O, MgCl_2_·6H_2_O, KCl, Mn(NO_3_)_2_·4H_2_O, NaCl, Ni(NO_3_)_2_·6H_2_O, Pb(NO_3_)_2_, LiNO_3_, and ZnCl_2_, all of which were dissolved in deionized water. Stock solutions (10 mM) of the tetrabutylammonium salts of CN^−^, SCN^−^, HPO_4_^2−^, Cl^−^, AcO^−^, I^−^, CO_3_^2−^, H_2_PO_4_^−^, NO_3_^−^, ClO_4_^−^, NO_2_^−^, SO_3_^2−^, OH^−^, SO_4_^2−^, and Br^−^ were prepared in DMSO. Stock solution (10 mM) of sodium salt of PO_4_^3−^ was prepared in H_2_O. UV–Vis absorption spectra of MH-5 were recorded using a quartz cuvette with a 1-cm path length over the 300–800 nm wavelength range, both in the presence and absence of the metal ions and the anions tested. Fluorescence emission spectra were obtained by exciting the samples at a wavelength of 530 nm and measuring emissions between 550 and 600 nm using a 3.0-mL cuvette. All spectroscopic measurements were conducted under consistent conditions at room temperature, with spectra collected 5 min after the addition of each metal ion.

### General procedure for fluorescence microscopy imaging in cultured cells

#### Cell culture

Colon adenocarcinoma cell line HT-29 was purchased from American Type Culture Collection. The HT-29 (human colorectal adenocarcinoma cell line) was cultured in RPMI (supplemented with 110 mg/L sodium pyruvate and 1 g/L glucose; Invitrogen, Gibco, Karlsruhe, Germany) containing 100 units/mL penicillin–streptomycin and gentamicin (0.01%). The L9-29 (epithelial cell line) was cultured in DMEM (4.5 g/L glucose; Gibco, Karlsruhe, Invitrogen, Germany) containing 100 units/mL penicillin/streptomycin and gentamicin (0.01%). All mediums were further supplemented with 10% fetal bovine serum (FBS) (Thermo Fisher Scientific). Cells were seeded in each well of uncoated 96-well microplates and incubated in a humidified atmosphere with 5% CO_2_ at 37 °C for 18 h.

#### Cell viability assay

The cytotoxicity of MH-5 in epithelial and cancer cell lines (L9-29 and HT-29) was evaluated using the Alamar Blue assay [55, 56]. Cells were cultured in DMEM and RPMI media at 37 °C in an atmosphere of 5% CO_2_. After each cell line reached a density of 1 × 10^4^ cells, they were harvested and seeded into a 96-well cell culture plate. Cells were then treated with 10 different concentrations (0.1 µM to 100 µM) of MH-5 and incubated for 18 h. Each sample was prepared in triplicate, with untreated cells as negative controls. After the 18-h incubation, Alamar Blue reagent (1:10, v/v) was added to each well and incubated for 4 h. Spectrophotometric measurements were taken at 570 nm and 600 nm. Cell viability for each sample was calculated, with the control wells set as 100%.

#### Fluorescence imaging

Cells cultured in RPMI were treated with 10 mM Li^+^ and CN^−^ ion solutions (2 µL; final concentration: 20 µM) dissolved in sterilized PBS (pH 7.4) and incubated at 37 °C for 30 min. After treatment, the cells were washed with PBS (2 mL × 3) to remove residual ions. RPMI (2 mL) was added to the cell culture, followed by treatment with an MH-5 solution (2 µL; final concentration: 20 µM) dissolved in DMSO-PBS buffer. Samples were incubated at 37 °C for 30 min. The culture medium was then removed, and the treated cells were washed with PBS (2 mL × 3) before observation. Fluorescence imaging was performed using a Leica fluorescence microscope. The cells were excited with a red fluorescent light laser at 590 nm, and emission was collected at 615 ± 25 nm.

## Results and discussions

**Scheme 1.**
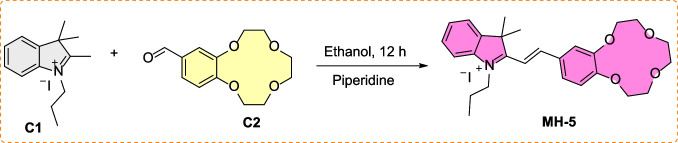
Synthesis of the sensor, MH-5

The synthesis of MH-5 was completed through a three-step reaction process. Initially, the starting compounds, 2,3,3-trimethyl-1-propyl-3H-indol-1-ium iodide and 2,3,5,6,8,9-hexahydrobenzo[b][1, 4, 7, 10]tetraoxacyclododecine-12-carbaldehyde [53, 54], were synthesized in the first and second steps. In the last step, these compounds were combined to yield MH-5. The structure of MH-5 was confirmed using NMR (both ^1^H and ^13^C NMR) and Q-TOF LC/MS mass spectrometry, with corresponding spectra provided in Figs. S1–S3 of the Electronic Supplementary Material. The synthetic pathway for MH-5 was illustrated in Scheme 1.

### Spectroscopic studies of MH-5 with cations

**Fig. 1:**
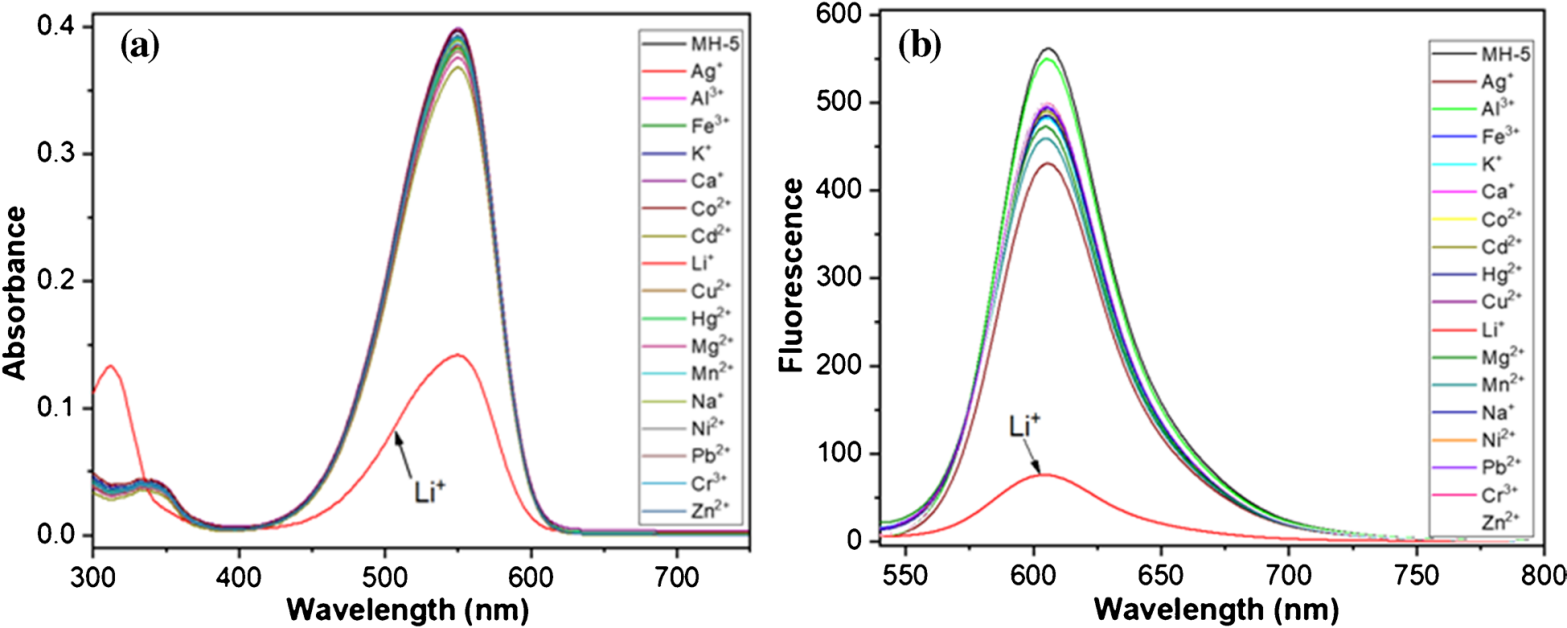
** a** Absorbance and **b** fluorescence spectra of 5.0 µM MH-5 to the metal ions tested (5.0 µM of Ag^+^, Al^3+^, Fe^3+^, K+, Ca^2+^, Co^2+^, Cd^2^^+^, Hg^2+^, Cu^2+^, Li^+^, Mg^2+^, Mn^2+^, Na^+^, Ni^2+^, Pb^2+^, Cr^3+^, and Zn^2+^) in DMSO-PBS buffer (10 mM, pH = 7.25, v/v, 1:9)

To determine the selectivity of MH-5, the color changes observable to the naked eye were initially tested by exposing MH-5 (5.0 μM) to various metal ions (5.0 μM), including Li^+^, Ag^+^, Al^3+^, Fe^3+^, K^+^, Ca^2+^, Co^2+^, Cd^2+^, Hg^2+^, Cu^2+^, Mg^2+^, Mn^2+^, Na^+^, Ni^2+^, Pb^2+^_,_ Cr^3+^, and Zn^2+^ in DMSO-PBS buffer (10 mM, pH 7.25, 1:9 v/v). Among all tested ions, only Li^+^ triggered a noticeable color shift in MH-5 from pink to pale pink (see Electronic Supplementary Material Fig. S4). Correspondingly, UV–Vis spectra of MH-5 with these metal ions were obtained under conditions similar as to those of the naked eye detection tests. MH-5 alone (5.0 μM) showed a peak absorption at 550 nm (ε = 7.80 × 10^4^ M^−1^ cm^−1^). However, when Li^+^ was added, the absorbance at 550 nm decreased significantly (ε = 2.58 × 10^4^ M^−1^ cm^−1^ at a 1:1 MH-5/Li^+^ ratio). In contrast, other ions including Ag^+^, Al^3+^, Fe^3+^, K^+^, Ca^2+^, Co^2+^, Cd^2+^, Hg^2+^, Cu^2+^, Mg^2+^, Mn^2+^, Na^+^, Ni^2+^, Pb^2+^_,_ Cr^3+^, and Zn^2+^ exhibited minimal responses, as shown by MH-5 analysis (Fig. 1a).

The metal ion selectivity of MH-5 was further evaluated using fluorescence spectroscopy. As shown in Fig. 1b, MH-5 exhibited a strong emission peak at 602 nm when excited at 530 nm, and the fluorescence quantum yield was calculated to be 0.096 [57]. Upon the addition of the metal ions tested, there was a slight quenching in fluorescence intensity at 602 nm; however, a substantial quenching effect (83%) was observed specifically with Li^+^, resulting color change under UV-lamb (see Electronic Supplementary Material Fig. S5). The quantum yield value also changed to 0.034. These findings from naked eye observation, absorption and fluorescence spectroscopy indicate that MH-5 serves as a highly selective colorimetric and on–off fluorescent sensor for detecting Li^+^.

The time response of a fluorescence sensor is a crucial parameter for assessing its practical applicability. The kinetics of the interaction between MH-5 and Li^+^ were examined in DMSO-PBS buffer solution (10 mM, pH 7.25, 1:9 v/v). As presented in Fig. S6 of the Electronic Supplementary Material, the emission intensity of MH-5 at 602 nm decreased rapidly upon the addition of 1.0 equivalent of Li^+^, reaching a minimum value within 2 min and remaining stable over long reaction times. This rapid response highlights the potential of MH-5 as an efficient sensor, providing time-saving advantages and enhancing its applicability in practical settings.

In order to understand the optimal working condition of MH-5 and MH-5/Li^+^, the effect of pH on selectivity was studied in the range of 3.5–10 pH. MH-5 showed no fluorescence response to hydrogen ions within a pH range of 3.5 to 9.0, indicating strong stability over a wide pH spectrum. In the presence of Li^+^, fluorescence quenching was observed at 602 nm, with the fluorescence signal remaining stable from pH 3.5 to 8.0. These findings suggest that MH-5 is suitable for Li^+^ detection within a pH range of 3.5 to 8.0, supporting its potential applicability in biological systems (see Electronic Supplementary Material Fig. S7).

**Fig. 2:**
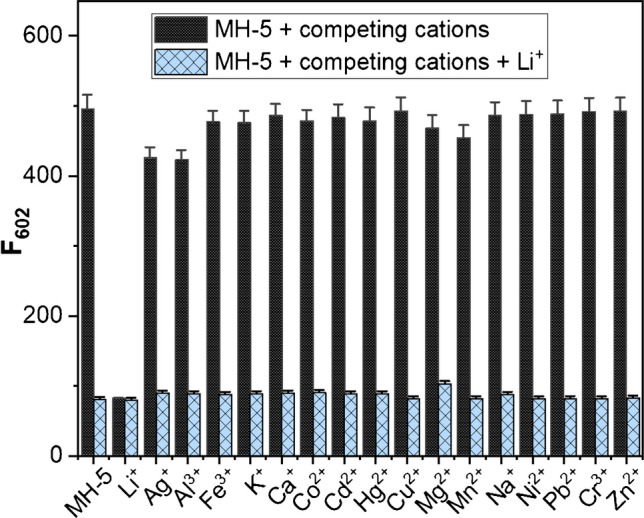
Fluorescence responses of 5.0 µM MH-5 to the presence of 50.0 µM metal ions tested (black bars) and the subsequent addition of Li^+^ (blue bars) in DMSO-PBS buffer (10 mM, pH = 7.25, v/v, 1:9); the bars represent the fluorescence intensity at 602 nm (excitation wavelength was 530 nm)

Competition studies were also performed to assess the selective sensing behavior of MH-5 toward Li^+^ in the presence of competing metal ions, including Ag^+^, Al^+3^, Fe^3+^, K^+^, Ca^2+^, Co^2+^, Cd^2+^, Hg^2+^, Cu^2+^, Li^+^, Mg^2+^, Mn^2+^, Na^+^, Ni^2+^, Pb^2+^_,_ Cr^3+^, and Zn^2+^. Upon adding 5.0 equivalents of these competing metal ions, a fluorescence quenching at 602 nm (indicated by blue bars) was observed following the subsequent addition of 5.0 equivalents of Li^+^. These findings indicate that MH-5 can function as an effective Li^+^ sensor even in the presence of various competing cations (Fig. 2).

### Spectroscopic studies of MH-5 with anions

**Fig. 3:**
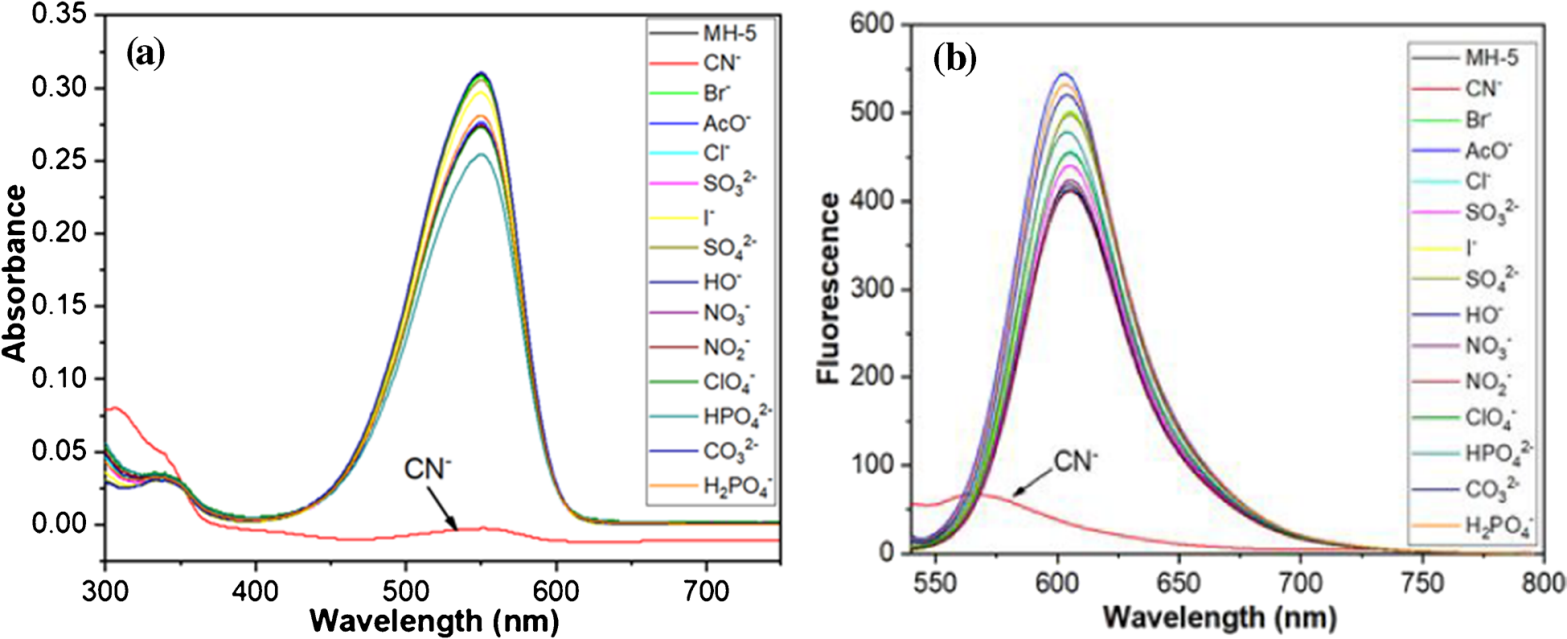
** a** Absorbance and **b** fluorescence spectra of 5.0 µM MH-5 to anions tested (5.0 µM of CN^−^, Br^−^, AcO^−^, Cl^−^, SO_3_^2−^, I^−^, SO_4_^2−^, OH^−^, NO_3_^−^, NO_2_^−^, ClO_4_^−^, HPO_4_^2−^, CO_3_^2−^, and H_2_PO_4_^−^) in DMSO-PBS buffer (10 mM, pH = 7.25, v/v, 1:9)

The remarkable photophysical characteristics of MH-5 were further examined using absorbance and emission spectroscopy in the presence of the following anions in their tetrabutylammonium salts from CN^−^, Br^−^, AcO^−^, Cl^−^, SO_3_^2−^, I^−^, SO_4_^2−^, OH^−^, NO_3_^−^, NO_2_^−^, ClO_4_^−^, HPO_4_^2−^, CO_3_^2−^, and H_2_PO_4_^−^ in DMSO-PBS buffer (10 mM, pH = 7.25, v/v, 1:9). As previously noted, MH-5 (5.0 µM) showed an absorption band with a molar absorptivity of ε = 7.80 × 10^4^ M^−1^ cm^−1^ at 550 nm. Upon the addition of CN^−^ to the MH-5 solution, the color changed from pink to colorless (see Electronic Supplementary Material Fig. S8), and the absorbance intensity at 550 nm nearly disappeared (Fig. 3a). In contrast, the color and UV–VİS spectrum of MH-5 remained largely unchanged in the presence of other tested anions.

The fluorescence spectra of MH-5 were obtained both in the absence and presence of CN^−^ in a DMSO-PBS buffer (10 mM, pH 7.25, 1:9 v/v). As illustrated in Fig. 3b, MH-5 (5.0 μM) exhibited strong fluorescence at 602 nm upon excitation at 530 nm, as mentioned above. While the addition of various anions resulted in a slight quenching of fluorescence intensity at 602 nm, a pronounced quenching effect (92%) was specifically observed with CN^−^, accompanied by a visible color change under UV light (see Electronic Supplementary Material Fig. S9). The quantum yield value also changed to 0.019. These results from naked eye observation, absorption, and fluorescence spectroscopy demonstrate that MH-5 functions as a highly selective colorimetric and on–off fluorescent sensor for CN^−^ detection.

The kinetic of the MH-5/CN^−^ interaction system was also studied in DMSO-PBS buffer solution (10 mM, pH 7.25, v/v, 1:9). As shown in Fig. S10 of the Electronic Supplementary Material, the emission intensity of MH-5 at 602 nm rapidly decreased upon the addition of 1.0 equivalent of CN^−^, reaching its minimum value within 5 min and remaining stable with extended reaction time. This property of MH-5 underscores its suitability as a highly efficient sensor, offering time-saving advantages and enhancing its utility for practical applications.

The effect of pH on CN^−^ detection by MH-5 was evaluated through fluorescence spectroscopy. Fluorescence spectra of MH-5, both in the presence and absence of CN^−^, were recorded across a pH range of 3.5 to 10 (see Electronic Supplementary Material Fig. S11). The sensor showed no response to hydrogen ions within a pH range of 3.5 to 9.0, indicating strong stability over a wide pH spectrum. In the presence of CN^−^, fluorescence quenching was observed at 602 nm, with the fluorescence signal remaining stable from pH 3.5 to 10.0. These findings suggest that MH-5 is suitable for CN^−^ detection within a pH range of 3.5 to 9.0, supporting its potential applicability in biological systems.

**Fig. 4:**
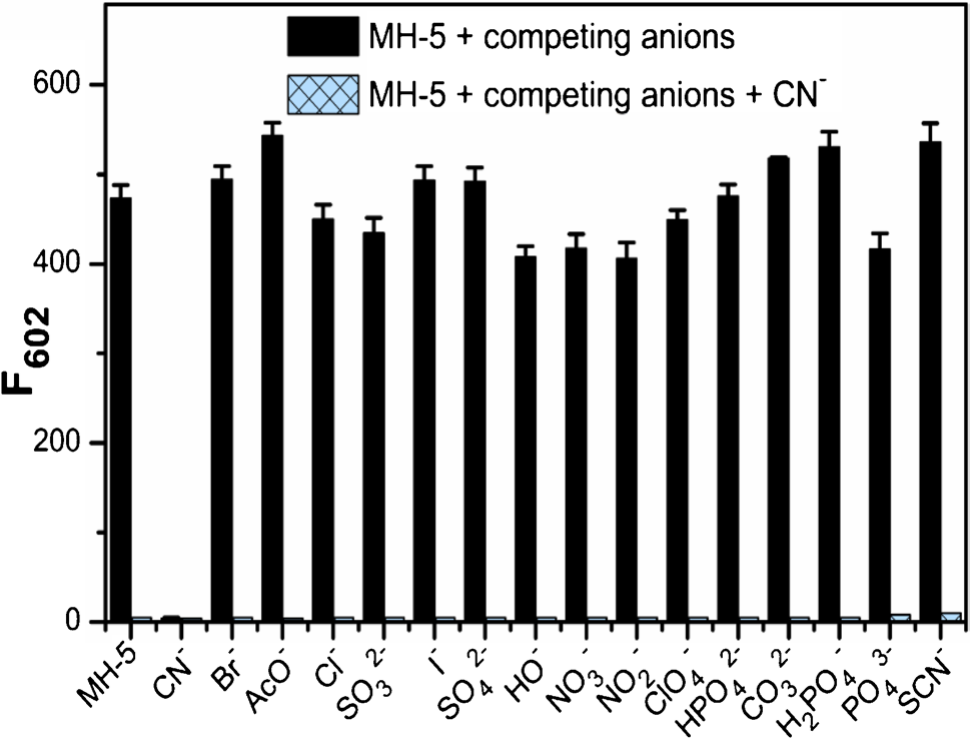
Fluorescence responses of 5.0 µM MH-5 to the presence of 50.0 µM anions tested (black bars) and the subsequent addition of CN^−^ (blue bars) in DMSO-PBS buffer (10 mM, pH = 7.25, v/v, 1:9); the bars represent the fluorescence intensity at 602 nm (excitation wavelength was 530 nm)

Competition studies were also performed to assess the selective sensing behavior of MH-5 toward CN^−^ in the presence of competing anions, including Br^−^, AcO^−^, Cl^−^, SO_3_^2−^, I^−^, SO_4_^2−^, OH^−^, NO_3_^−^, NO_2_^−^, ClO_4_^−^, HPO_4_^2−^, CO_3_^2−^, H_2_PO_4_^−^, PO_4_^3−^, and SCN^−^. Upon adding 5.0 equivalents of these competing anions, a fluorescence quenching at 602 nm (indicated by blue bars) was observed following the subsequent addition of 5.0 equivalents of CN^−^. These findings indicate that MH-5 can function as an effective CN^−^ sensor even in the presence of various competing anions (Fig. 4).

### Proposed binding mode and sensing mechanisms

To elucidate the binding mode of MH-5 with Li^+^ and CN^−^, the binding stoichiometries for MH-5 with Li^+^ and with CN^−^ were initially examined. Various analytical techniques were employed to accurately determine the stoichiometry of interactions of MH-5 with Li^+^ and CN^−^, respectively. Job’s plot, derived from UV–Vis spectroscopic measurements, exhibited a maximum absorption when the molar fraction of Li^+^ and CN^−^ reached approximately 0.5 (see Electronic Supplementary Material Fig. S12 and Fig. S13). This result suggests a 1:1 binding stoichiometry between MH-5 and both Li^+^ and CN^−^.

**Fig. 5:**
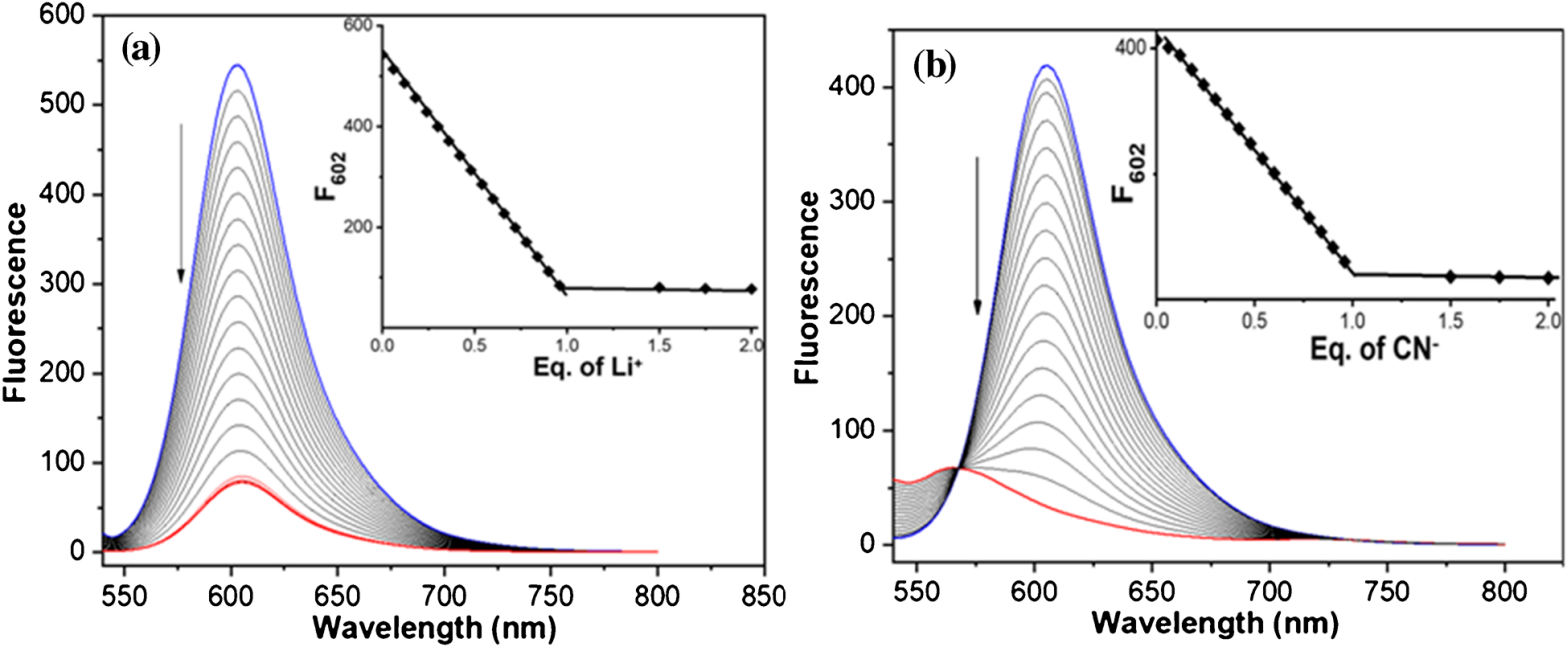
Fluorescence titration of 5.0 µM MH-5 with increasing concentrations (0, 0.3, 0.6, 0.9, 1.2, 1.5, 1.8, 2.1, 2.4, 2.7, 3.0, 3.3, 3.6, 3.9, 4.2, 4.5, 4.8, 5.0, 7.5, and 10 µM, respectively) in DMSO-PBS buffer (10 mM, pH = 7.25, v/v, 1:9) of Li^+^ (**a**) and CN^−^ (**b**)

UV–Vis titration and fluorescence titration were also applied to study the binding stoichiometry between MH-5 and Li^+^ and CN^−^. As depicted in Fig. S14 and S15 of the Electronic Supplementary Material, the absorption intensity of MH-5 (5.0 µM) in DMSO-PBS buffer (10 mM, pH = 7.25, v/v, 1:9) at 550 nm gradually decreased upon the addition of Li^+^ and CN^−^ at varying concentrations, reaching saturation upon the addition of 1.0 equivalent of each ion. This behavior suggests a 1:1 stoichiometric interaction between MH-5 and both Li^+^ and CN^−^. Furthermore, clear isosbestic points were observed at 337 nm for Li^+^ and 354 nm for CN^−^, indicating a shift from the free form of MH-5 to the complexed forms MH-5 + Li^+^ and MH-5 + CN^−^as binding interactions occur [58]. Fluorescence titration experiments also supported the 1:1 stoichiometry between MH-5 and both Li^+^ and CN^−^. As seen in Fig. 5a and 5b, the titration curve (a plot from fluorescence intensities versus Li^+^ or CN^−^ at various concentrations) decreased linearly, reaching saturation at 1:1 ratio of MH-5 between Li^+^ and CN^−^. This outcome is consistent with a 1:1 binding stoichiometry for both ions. MALDI-TOF mass results also supported a 1:1 ratio between MH-5 and CN^−^ (see Electronic Supplementary Material Fig. S16).

**Fig. 6:**
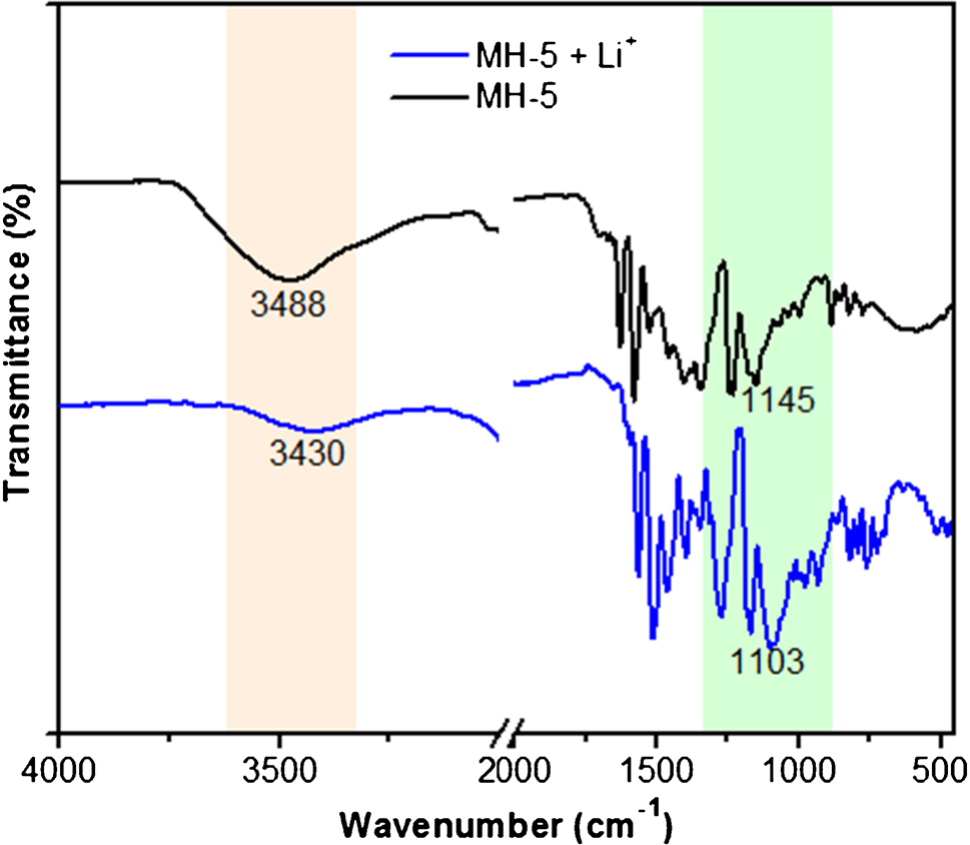
FT-IR spectra of MH-5 (black line) and MH-5 + Li^+^ (blue line)

FT-IR spectroscopy was used to elucidate the sensing mechanism of MH-5 for Li^+^ ions (Fig. 6). Upon addition of Li^+^, the peaks of -OH and -C–O–C at 3488 cm^−1^ and 1103 cm^−1^, respectively, shifted. These findings indicate that Li^+^ ion interacts with the crown ether portion of MH-5 [59].

**Fig. 7:**
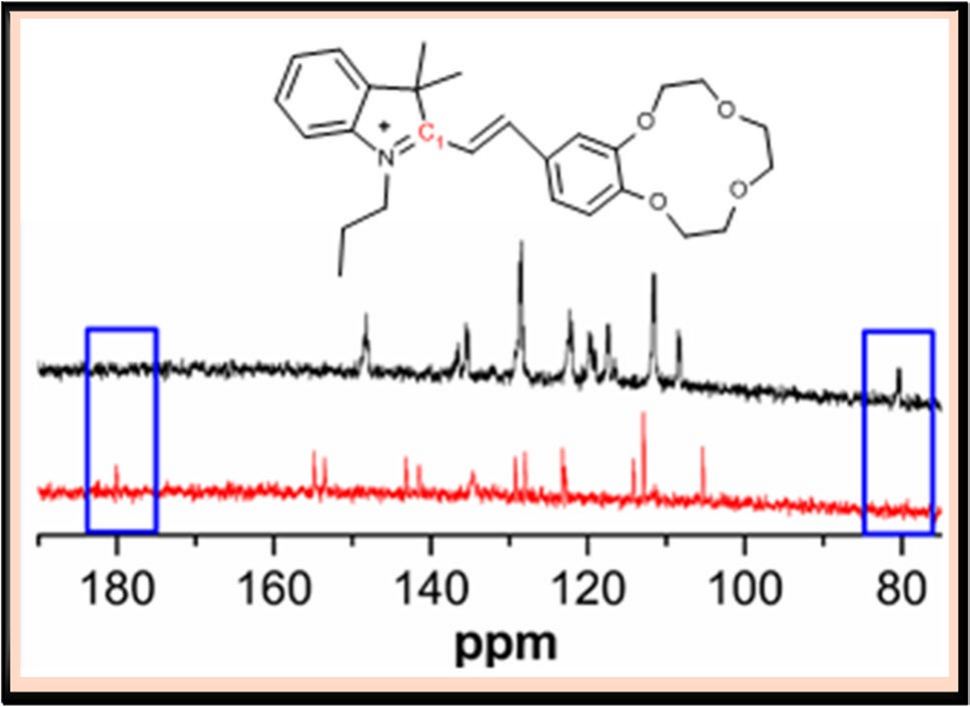
^13^C NMR spectra of MH-5 (red line) and in the presence of CN^−^ (black line)

The sensing mechanism between MH-5 and CN^−^ was elucidated through ^13^C NMR titration experiments. In these experiments, CN^−^ (dissolved in DMSO-d6) was added to a DMSO-d6 solution of MH-5, allowing for detailed observation of binding interactions. As illustrated in Fig. 7, the peak at 180 ppm belonging to the carbon numbered as C1 in the molecule shifted to 81.2 ppm after CN^−^ was added. This is probably due to the conversion of C1 from sp^2^ hybridization to sp^3^ hybridization [60]. Moreover, a new peak at 118.2 ppm appeared, corresponding to CN^−^ [61]. These results indicate a nucleophilic attack by CN^−^ on the carbon atom (C1) of the indoline moiety [62, 63].

We also studied the reversibility of the binding of Li^+^ and CN^−^ by MH-5 in DMSO-PBS buffer (10 mM, pH = 7.25, v/v, 1:9), respectively. Ethylenediaminetetraacetic acid (EDTA) was used to remove Li^+^ ion from the MH-5 + Li^+^ [51]. As depicted in Fig. S17 of the Electronic Supplementary Material, the addition of EDTA led to a rapid increase in the fluorescence peak at 602 nm, indicating that MH-5 can detect Li^+^ through a reversible mechanism. On the other hand, trifluoroacetic acid (TFA) was used to remove the CN^−^ ion from MH-5 + CN^−^ [64]; however, as shown in Fig. S18 of the Electronic Supplementary Material, no significant change was observed in the fluorescence spectrum of MH-5 + CN^−^. These results suggest an irreversible interaction between MH-5 and CN^−^.

**Scheme 2.**
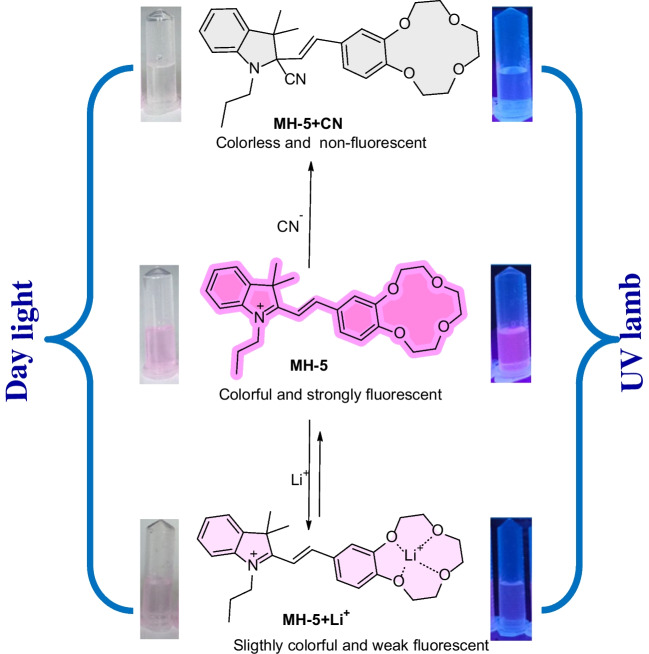
Proposed response mechanisms of MH-5 toward Li^+^ and CN^−^

Based on the results obtained, the binding mechanisms of MH-5 between Li^+^ and CN^−^ were proposed as shown in Scheme 2. CN^−^ detection was achieved through its nucleophilic addition to the indolium group of the sensor [65, 66], while Li^+^ was detected by its coordination with oxygen atoms in the crown ether moiety [47, 67]. In both detection mechanisms, the binding interactions with Li^+^ and CN^−^ induced fluorescence quenching in the fluorescence of MH-5.

**Fig. 8:**
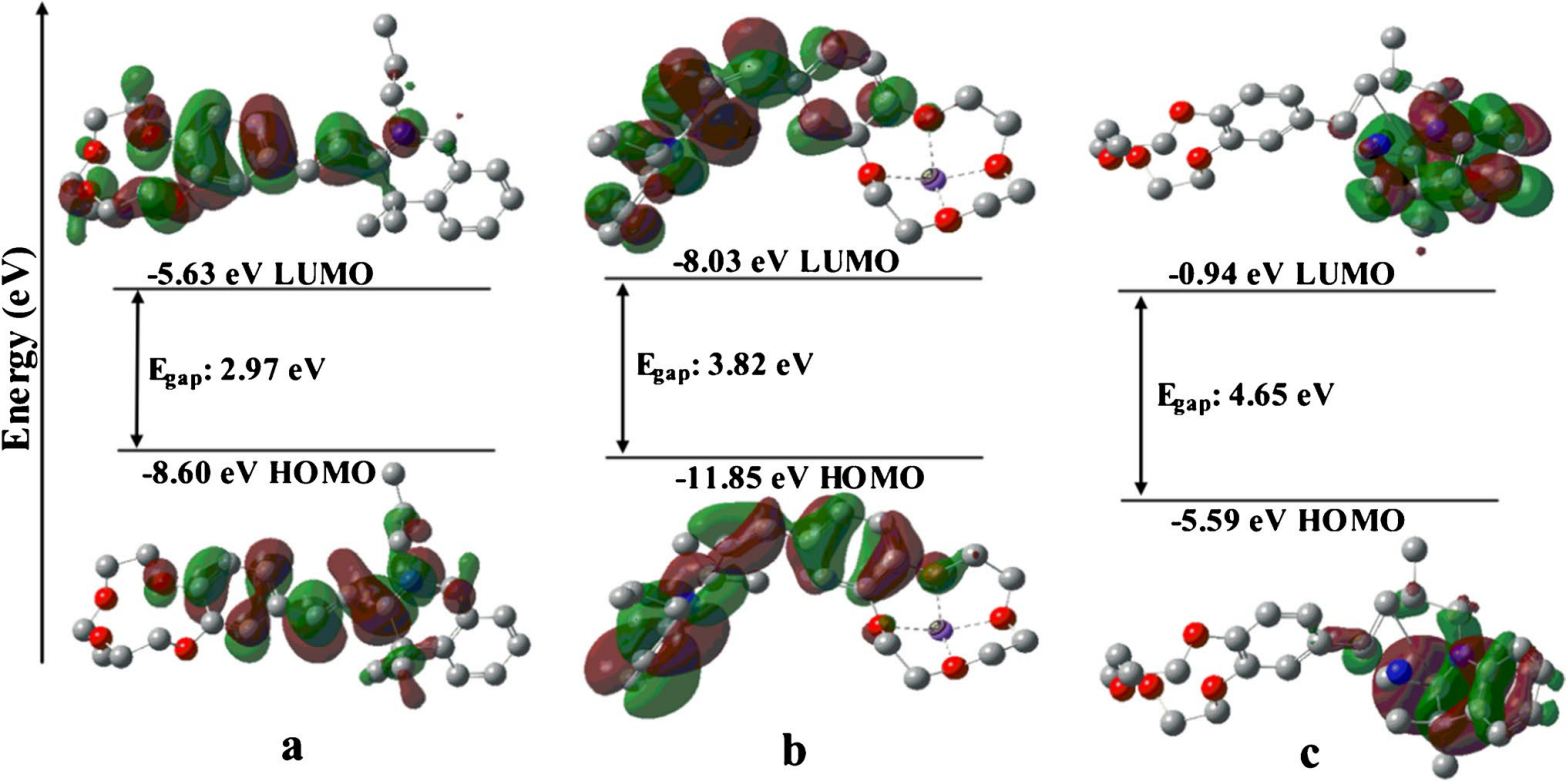
Energy diagrams of HOMO and LUMO orbitals: **a** MH-5, **b** MH-5 + Li^+^, **c** MH-5 + CN^−^

To further confirm the binding mechanism between MH-5 and Li^+^/CN^−^, optimized structures of MH-5, MH-5 + Li^+^, and MH-5 + CN^−^ were obtained by density functional theory (DFT) calculations at the B3LYP/6-311G (d,p) level using the Gaussian 09 program [68]. As seen in Fig. 8, the orbital spatial distributions were found as − 8.60 eV/ − 5.63 eV (HOMO → LUMO) for MH-5, − 11.83 eV/ − 8.03 eV (HOMO → LUMO) for MH-5 + Li^+^, and − 5.59 eV/ − 0.94 eV (HOMO-1 → LUMO) for MH-5 + CN^−^. The energy gaps of MH-5, MH-5 + Li^+^, and MH-5 + CN^−^ were found as 2.97 eV, 3.82 eV, and 4.65 eV, respectively. The energy gaps of MH-5 + Li^+^ and MH-5 + CN^−^ were higher than that of MH-5. The increasing energy gap between the HOMO and LUMO induces a blue shift in the absorbance wavelength for both Li^+^ and CN^−^ [37, 69]. These results support the proposed mechanisms illustrated in Scheme 2.

### Detection limit studies

The detection limits for MH-5 with Li^+^ and CN^−^ ions were determined through absorption and fluorescence analyses across a concentration range of MH-5/Li^+^ or CN^−^ from 0.1 to 5.0 µM. Calibration curves were generated by applying linear regression to the relationship between Li^+^ or CN^−^ concentrations and the corresponding absorption or fluorescence intensities. Detection limits were then calculated using the formula 3*σ*/*k* [70–72], where *σ* represents the standard deviation of blank signals from MH-5 (with *n* = 10) and *k* denotes the slope of the calibration curves.

As depicted in Fig. S19 and Fig. S20 of the Electronic Supplementary Material, the calibration curves exhibit strong linear correlations between CN^−^ concentrations and both absorbance and fluorescence intensities. The correlation coefficients are high, with *R*^2^ = 0.9971 for the absorbance measurements (*y* = − 0.063*x* + 0.310) and *R*^2^ = 0.9979 for the fluorescence measurements (*y* = − 76.697*x* + 430.04). The calculated detection limits were 0.355 µM for absorbance-based analysis and 0.154 µM for fluorescence-based analysis. Absorbance and fluorescence-based determination limit calculations were also performed for Li^+^. As shown in Fig. S21 and Fig. S22 of the Electronic Supplementary Material, the calibration curves show good linear relationships between Li^+^ concentration and absorbance/fluorescence intensities, with good correlation coefficients (*R*^2^ = 0.9982, *y* = − 0.0512*x* + 0.3914 from the absorbance measurements, *R*^2^ = 0.9991, *y* = − 94.41*x* + 541.7 from the fluorescence measurements). The detection limits were determined to be 0.189 µM for the absorbance-based study and to be 0.150 µM for the fluorescence-based study. The calculated detection limits for both Li^+^ and CN^−^ are below the concentration thresholds for these ions permitted in drinking water [4, 73].

### *Biological application of MH-5 in detecting Li*^+^*and CN*^*−*^

**Fig. 9:**
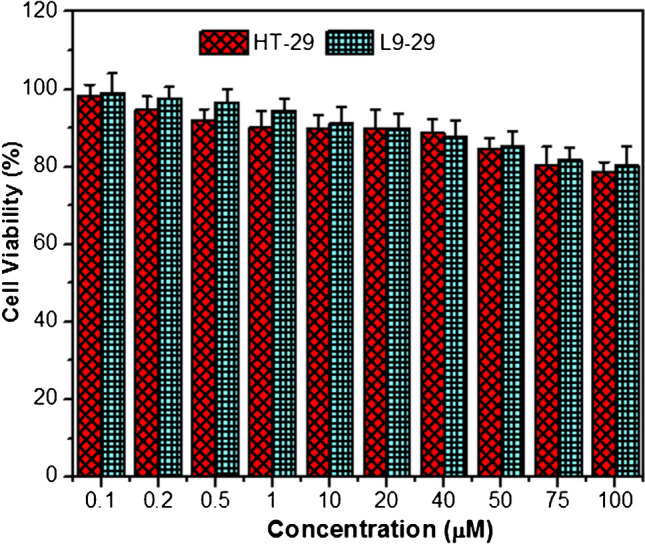
Cell viability (%) of the MH-5 on HT-29 and L9-29 cell lines. The error bars represent the mean ± SE of three independent measurements, with significance set at *p* < 0.05

The cytotoxic effect of the MH-5 material at different concentrations on HT-29 and L9-29 cell lines was investigated using the Alamar Blue assay [55, 56]. The MH-5 material induced 21.24% and 19.65% cell death rates in cancerous and healthy cell lines, respectively (Fig. 9). These cell death rates suggest that the material has relatively low or moderate toxicity. Cell death around 20% does not indicate high toxicity; however, it still shows an effect that warrants attention.

**Fig. 10:**
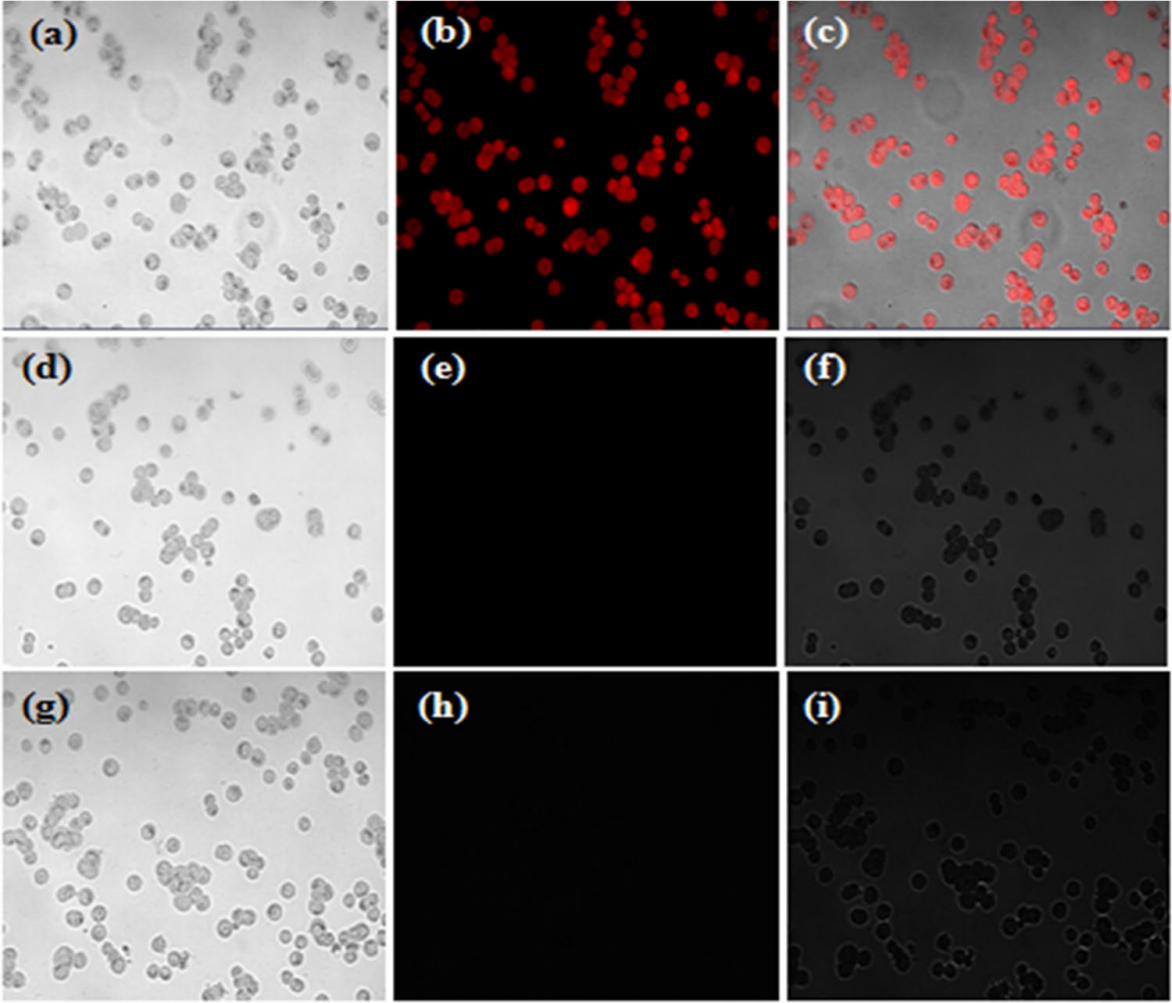
Fluorescence images of Li^+^ and CN^−^ with MH-5 in HT-29 cell lines. **a** Bright-field image of HT-29 cells incubated with MH-5 (10 µM). **b** The fluorescence image of HT-29 cells with MH-5. **c** Merged image of **a** and **b**. **d** Bright-field image of HT-29 cells pre-incubated with LiNO_3_ (10 μM) and then incubated with MH-5 (10 μM). **e** The fluorescence image of HT-29 cells with Li^+^ and MH-5. **f** Merged image of **d** and **e**. **g** Bright-field image of HT-29 cells pre-incubated with CN^−^ (10 μM) and then incubated with MH-5 (10 μM). **h** The fluorescence image of HT-29 cells with CN^−^ and MH-5. **i** Merged images of **g** and **h**

Even though we tested the toxicology of MH-5 on two different cell lines, HT-29 was chosen for cell imaging. The ability to detect Li^+^ in live HT-29 was investigated using fluorescence microscopy. Live HT-29 cells incubated with 10 µM MH-5 showed weak fluorescence (Fig. 10b). From the merged image of the cell lines, it can be seen that MH-5 molecules can easily pass through the cell membrane (Fig. 10c) Then, the cells were loaded with Li^+^. For Li^+^ loading conditions, live HT-29 cells were incubated with 10 µ LiNO_3_ at 37 °C for 4 h and then 10 µM MH-5 was added to the culture medium and incubated at 37 °C for 30 min. As seen in Fig. 10e, HT-29 cells incubated with Li^+^ did not show fluorescence. The same experiments were carried out for CN^−^ ions. The cells were loaded with CN^−^. For CN^−^ loading conditions, live HT-29 cells were incubated with 10 µ CN^−^ at 37 °C for 4 h, and then 10 µM MH-5 was added to the culture medium and incubated at 37 °C for 30 min. As seen in Fig. 10h, HT-29 cells incubated with CN^−^ did not show fluorescence. In general, it can be concluded that Li^+^ and CN^−^ ions in living cells are important molecules in selectivity by inhibiting the fluorescence properties of cell lines that are easily stained by the MH-5 chemosensor without damaging the cell lines.

### Comparison with previously reported sensors

The sensing ability of the present sensor toward CN^−^ and Li^+^ ions was also compared with others reported sensors in the literature, which is shown in Table S1. From the comparison, it is evident that the present sensor MH-5 has several advantages such as simple synthesis route, fast response time, low detection limit, and bio-imaging applications.

## Conclusion

In this study, a novel fluorescence sensor, MH-5, was successfully developed for the detection of Li^+^ and CN^−^ ions. The sensor was synthesized through a three-step process: a hemicyanine moiety was coupled with a crown ether moiety. Upon interaction with Li^+^ and CN^−^ ions, MH-5 exhibited distinct color changes from pink to pale pink and pink to colorless, respectively. The sensor demonstrated high selectivity and sensitivity, with significant absorbance and fluorescence quenching effects specific to Li^+^ and CN^−^, even in the presence of other tested cations and anions. Stoichiometric analysis revealed a 1:1 binding ratio between MH-5 and both Li^+^ and CN^−^ ions. The sensing mechanisms were also investigated using several analytical methods. The sensing mechanisms were verified with the NMR studies for CN^−^ and the FT-IR studies for Li^+^. For practical applications, MH-5 was further tested in living cells, where it efficiently permeated cell membranes and successfully visualized Li^+^ and CN^−^ ions. These findings suggest that MH-5 offers a promising approach for selective and effective detection of Li^+^ and CN^−^ ions, with potential applicability in bio-imaging and diagnostic fields.

## Supplementary Information

Below is the link to the electronic supplementary material.

ESM 1 (DOC 16.6 MB)

## Data Availability

All data are provided in full in the results section of this paper and supplementary information accompanying this paper.
