# Supplementary material for: A hemicyanine-based dual-responsive fluorescent sensor for the detection of lithium and cyanide ions: application in living cells
Source: Anal Bioanal Chem. 2025 Apr 3;417(14):3127–39. doi: 10.1007/s00216-025-05852-w (PMC12103329; doi:10.1007/s00216-025-05852-w)
Supplement: Supplementary file 1 — (DOC 16.6 MB) [file 216_2025_5852_MOESM1_ESM.doc]

SUPPORTING INFORMATION FOR

**A hemicyanine based dual-responsive fluorescent sensor for the detection of lithium and cyanide ions: application in living cells**

Ziya Aydin^a*^, Mukaddes Keskinates^b^, Esra Armagan^c^, Bahar Yilmaz Altinok^d^, Mevlut Bayrakci^d**^

^a^Karamanoglu Mehmetbey University, Vocational School of Technical Sciences, 70100 Karaman, Turkey

^b^Karamanoglu Mehmetbey University, Kazım Karabekir Vocational School, Department of Environmental Protection Technologies, 70100 Karaman, Turkey

^c^Karamanoglu Mehmetbey University, Ermenek Uysal and Hasan Kalan Health Services Vocational School, Department of Pharmacy Services, 70400 Karaman, Turkey

^d^Karamanoglu Mehmetbey University, Faculty of Engineering, Department of Bioengineering, 70200, Karaman, Turkey

^*^Corresponding Author. Email: [ziyaaydin@kmu.edu.tr](mailto:mbayrakci@kmu.edu.tr), Tel: +903382264543, Fax: +903382262023

^**^Corresponding Author. Email: [mevlutbayrakci@gmail.com](mailto:mevlutbayrakci@gmail.com), [mbayrakci@kmu.edu.tr](mailto:mbayrakci@kmu.edu.tr)

**Scheme S1.** Synthesis route for MH-5


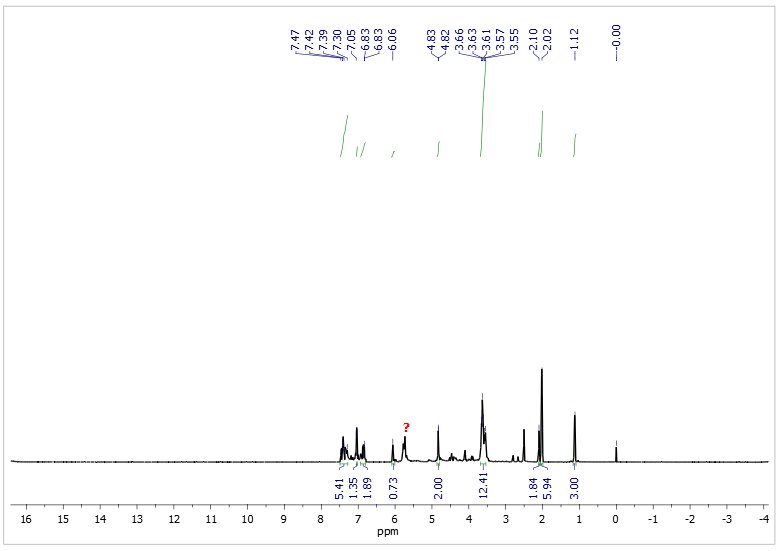


**Fig. S1.** ^1^H NMR of MH-5


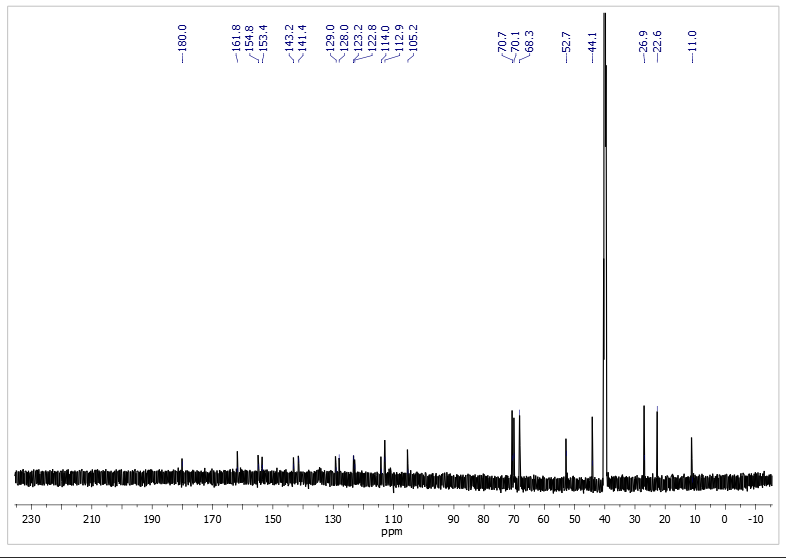
**Fig. S2.** ^13^C NMR of MH-5

**
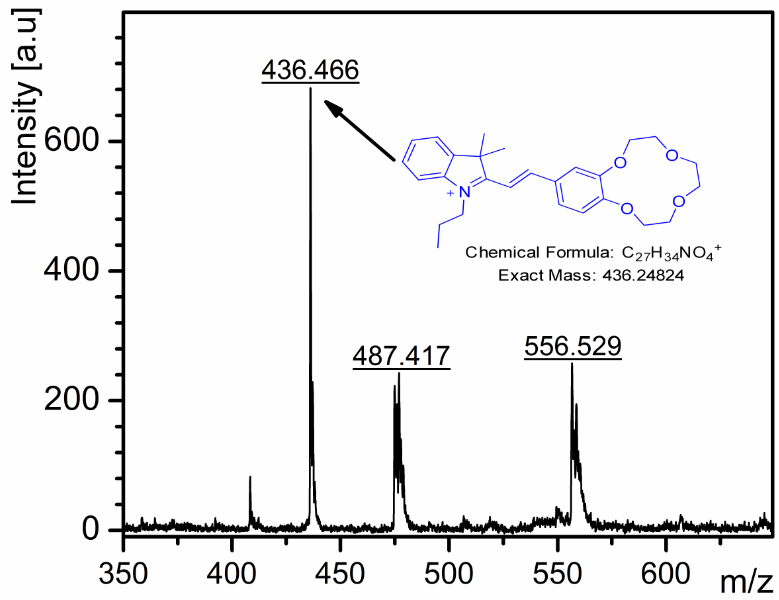
**

**Fig. S3.** MALDI-TOF spectrum of MH-5

**Fig. S4**. Color changes of 5.0 μM MH-5 with various metal ions in day light (5.0 μM for 1: Zn^2+^, 2: Cr^3+^, 3: Ni^2+^, 4: Hg^2+^, 5: Fe^3+^, 6: Mn^2+^, 7: Ag^+^, 8: Pb^2+^, 9: Cd^2+^, 10: Cu^2+^, 11: Li^+^, 12: Co^2+^, 13: Na^+^, 14: K^+^, 15: Mg^2+^, 16: Ca^2+^, 17: Al^3+^) in DMSO-PBS buffer (10 mM, pH=7.25, v/v, 1:9).

**Fig. S5**. Color changes of 5.0 μM MH-5 with various metal ions under UV light (5.0 μM for 1: Zn^2+^, 2: Cr^3+^, 3: Ni^2+^, 4: Hg^2+^, 5: Fe^3+^, 6: Mn^2+^, 7: Ag^+^, 8: Pb^2+^, 9: Cd^2+^, 10: Cu^2+^, 11: Li^+^, 12: Co^2+^, 13: Na^+^, 14: K^+^, 15: Mg^2+^, 16: Ca^2+^, 17: Al^3+^) in DMSO-PBS buffer (10 mM, pH=7.25, v/v, 1:9).


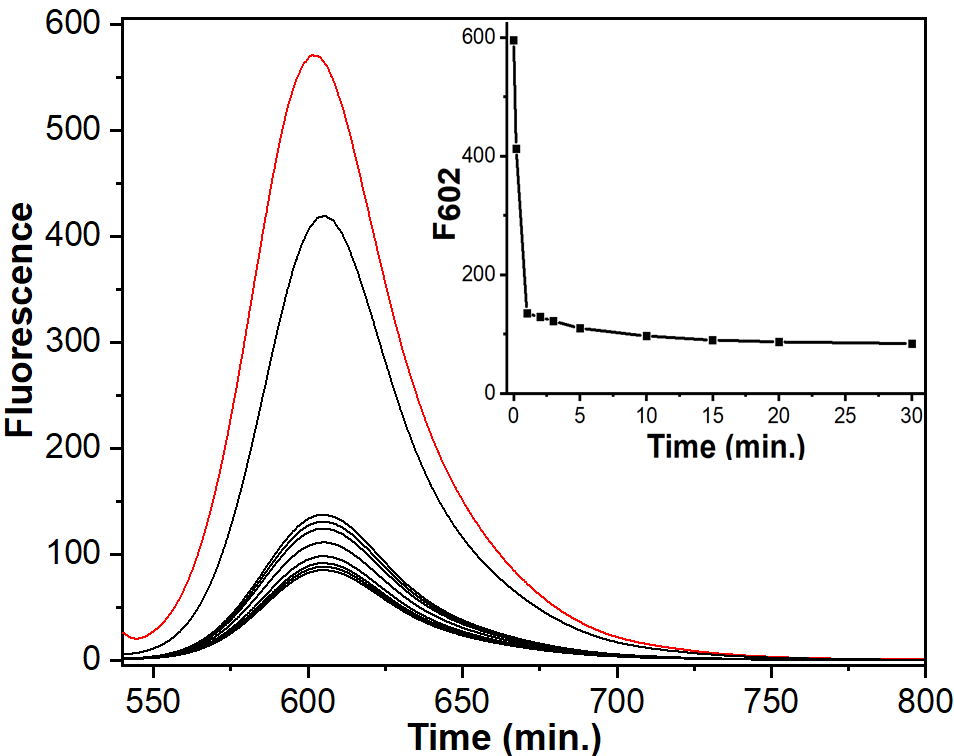


**Fig. S6.** Fluorescence spectra of MH-5+Li^+^ collected at different reaction time intervals (0, 1, 2, 3, 4, 5, 10, 15, 20, 25, 30 minutes) in DMSO-PBS buffer (10 mM, pH=7.25, v/v, 1:9).


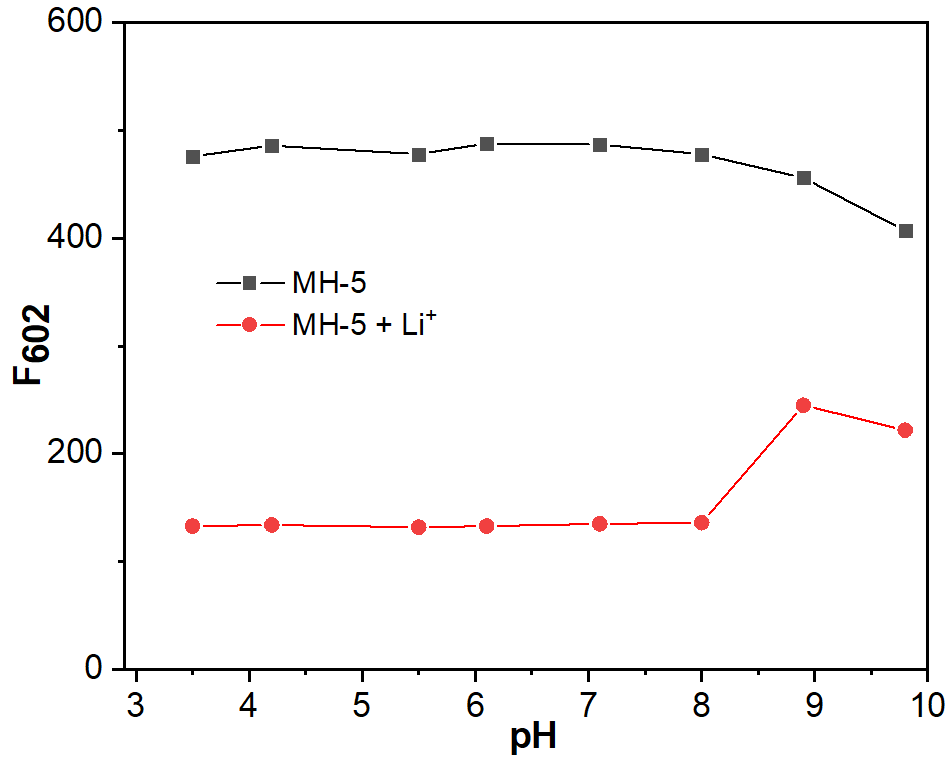


**Fig. S7.** Effect of pH on the fluorescence intensity of MH-5 and MH-5+Li^+^ at various pH values (DMSO/Buffered solution (9/1, v/v solution).

**Fig. S8**. Color changes of 5.0 μM MH-5 with various anions in day light (5.0 μM for 1: Cl^-^, 2: HO^-^, 3: I^-^, 4: CO_3_^2-^, 5: CN^-^, 6: HPO_4_^2-^, 7: AcO^-^, 8: NO_3_^-^, 9: H_2_PO_4_^-^, 10: NO_2_^-^, 11: ClO_4_^-^, 12: SO_4_^2-^, 13: Br^-^, 14: S^2-^ and 15: SO_3_^2-^ in DMSO-PBS buffer (10 mM, pH=7.25, v/v, 1:9).

**Fig. S9**. Color changes of 5.0 μM MH-5 with various anions under UV light (5.0 μM for 1: Cl^-^, 2: HO^-^, 3: I^-^, 4: CO_3_^2-^, 5: CN^-^, 6: HPO_4_^2-^, 7: AcO^-^, 8: NO_3_^-^, 9: H_2_PO_4_^-^, 10: NO_2_^-^, 11: ClO_4_^-^, 12: SO_4_^2-^, 13: Br^-^, 14: S^2-^ and 15: SO_3_^2-^ in DMSO-PBS buffer (10 mM, pH=7.25, v/v, 1:9).


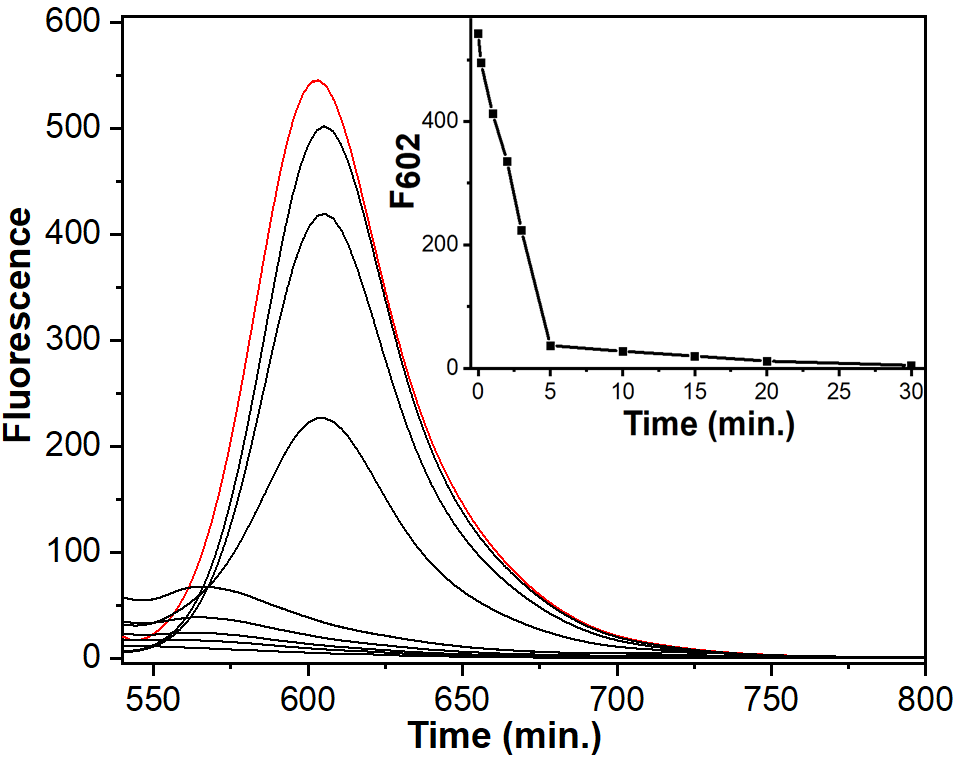


**Fig. S10**. Fluorescence spectra of MH-5+CN^-^ collected at different reaction time intervals (0, 1, 2, 3, 4, 5, 10, 15, 20, 25, 30 minutes) in DMSO-PBS buffer (10 mM, pH=7.25, v/v, 1:9).


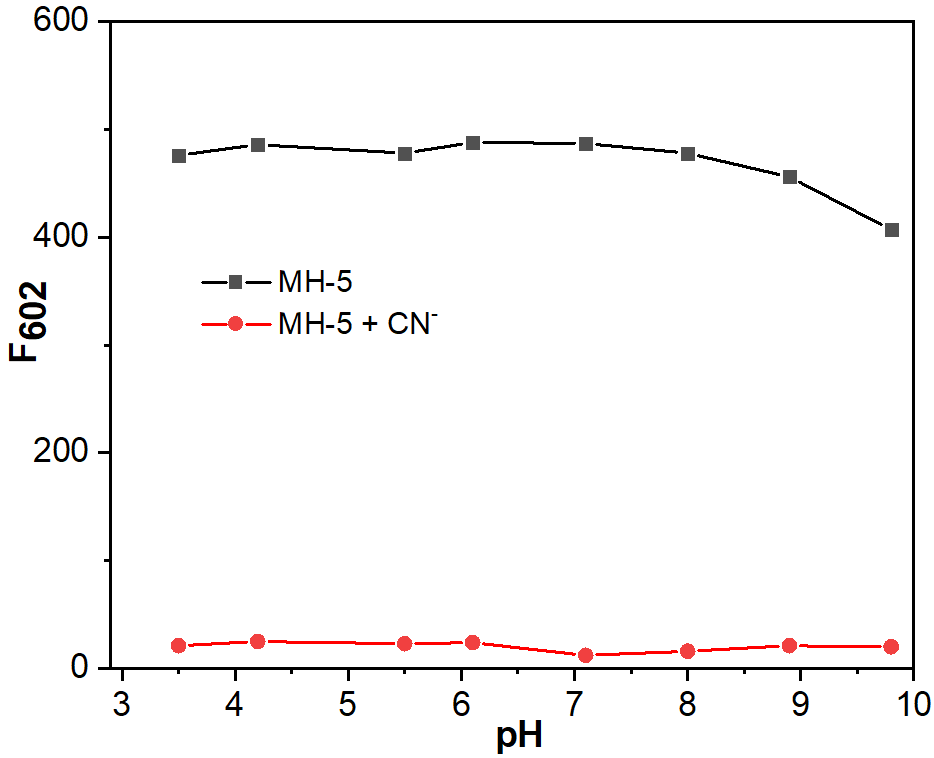


**Fig. S11**. Effect of pH on the fluorescence intensity of MH-5 and MH-5+CN^-^ at various pH values (DMSO/Buffered solution (9/1, v/v solution).


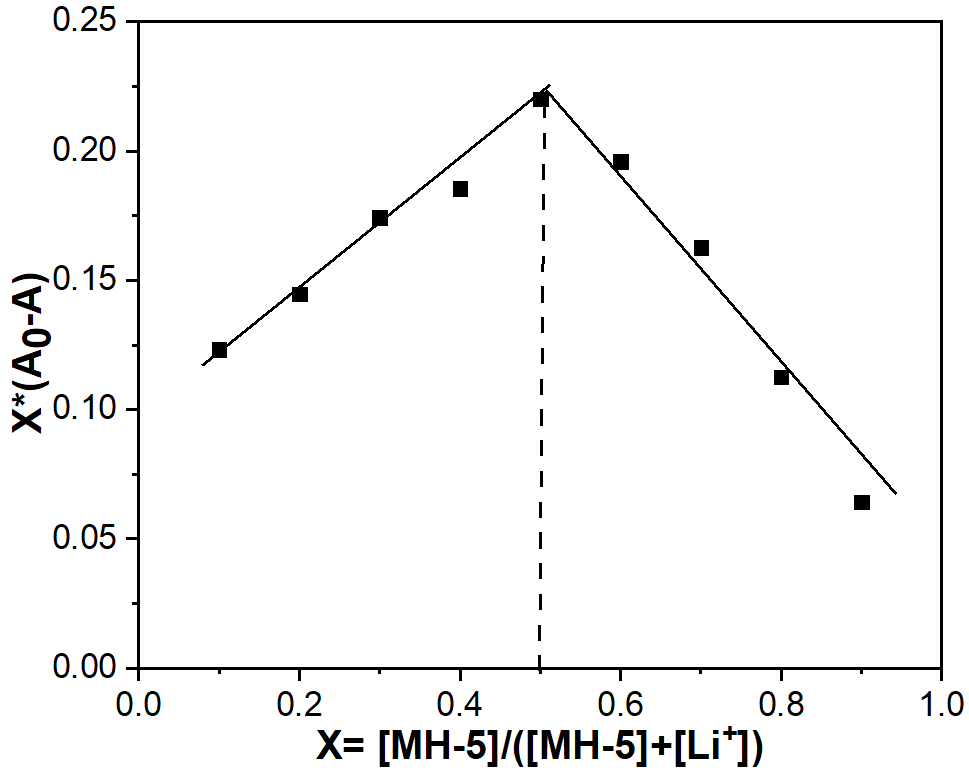


**Fig. S12.** Job’s plot for the interaction between MH-5 and Li^+^.


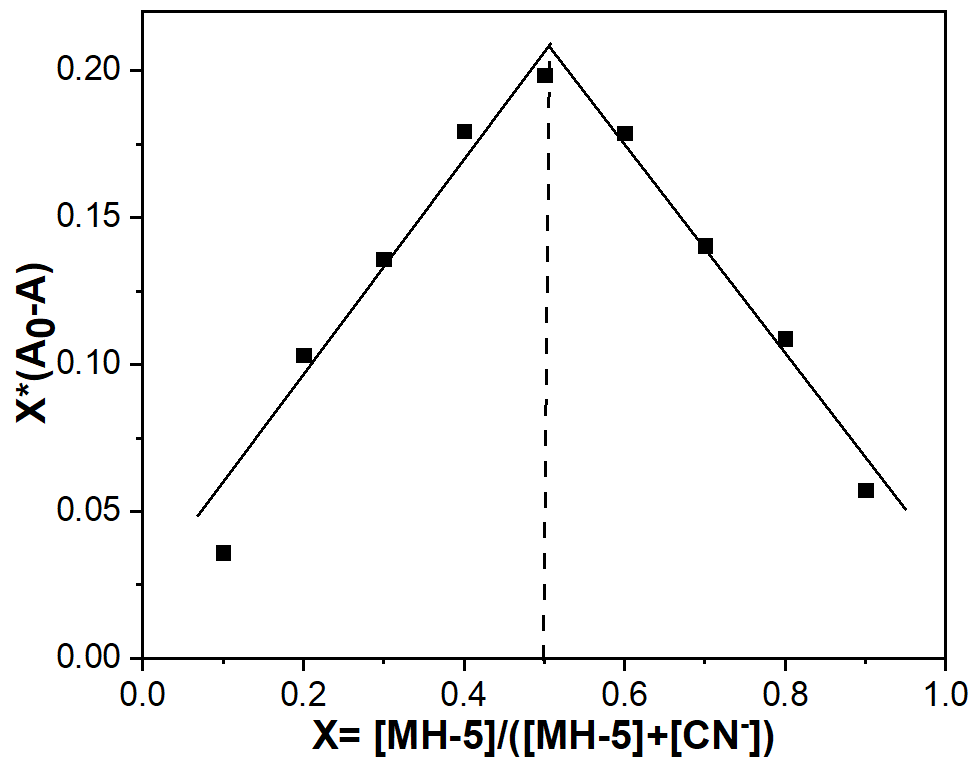


**Fig. S13.** Job’s plot for the interaction between MH-5 and CN^-^.


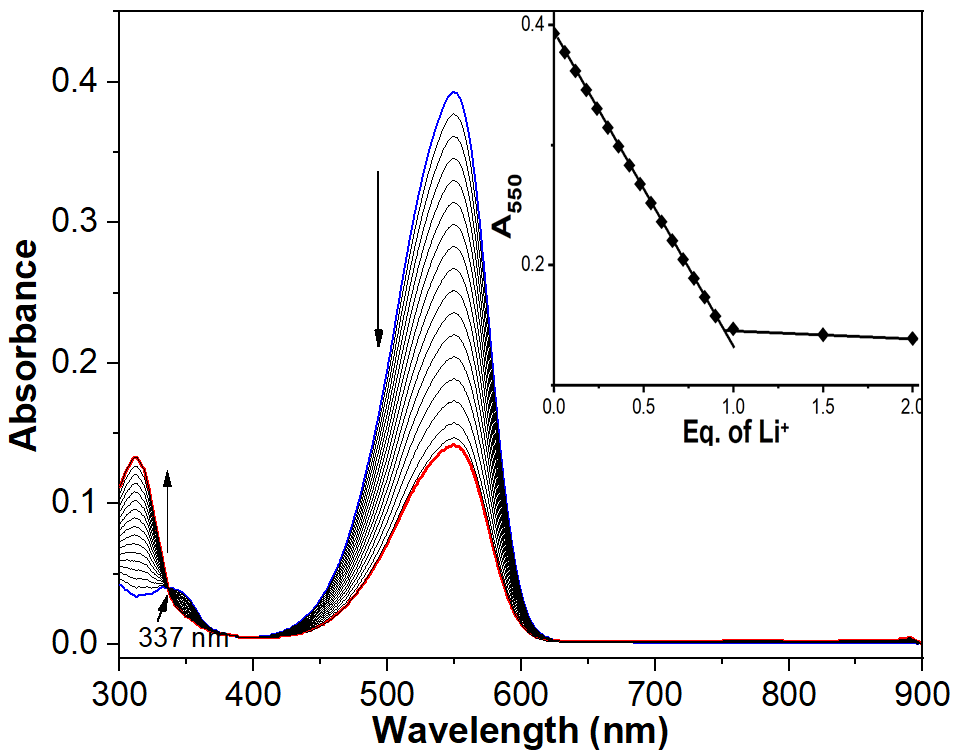


**Fig. S14.** Absorbance titration of 5.0 µM MH-5 with increasing concentrations of Li^+^ (0, 0.3, 0.6, 0.9, 1.2, 1.5, 1.8, 2.1, 2.4, 2.7, 3.0, 3.3, 3.6, 3.9, 4.2, 4.5, 4.8, 5.0, 7.5, and 10 µM, respectively) in DMSO-PBS buffer (10 mM, pH=7.25, v/v, 1:9).


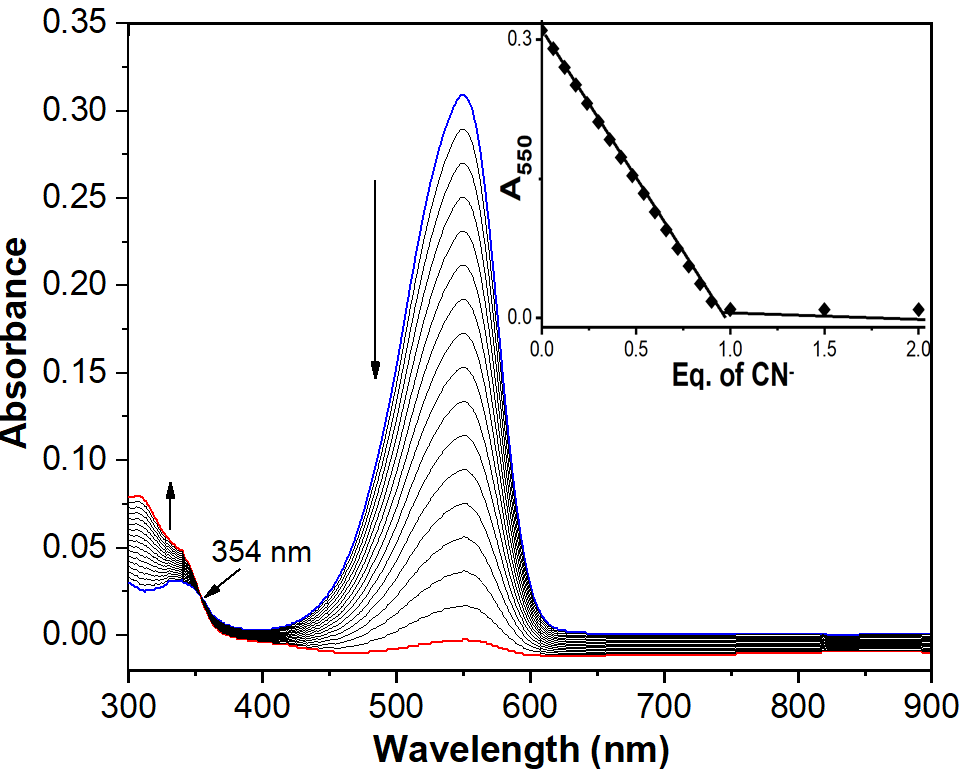


**Fig. S15.** Absorbance titration of 5.0 µM MH-5 with increasing concentrations of Li^+^ (0, 0.3, 0.6, 0.9, 1.2, 1.5, 1.8, 2.1, 2.4, 2.7, 3.0, 3.3, 3.6, 3.9, 4.2, 4.5, 4.8, 5.0, 7.5, and 10 µM, respectively) in DMSO-PBS buffer (10 mM, pH=7.25, v/v, 1:9).


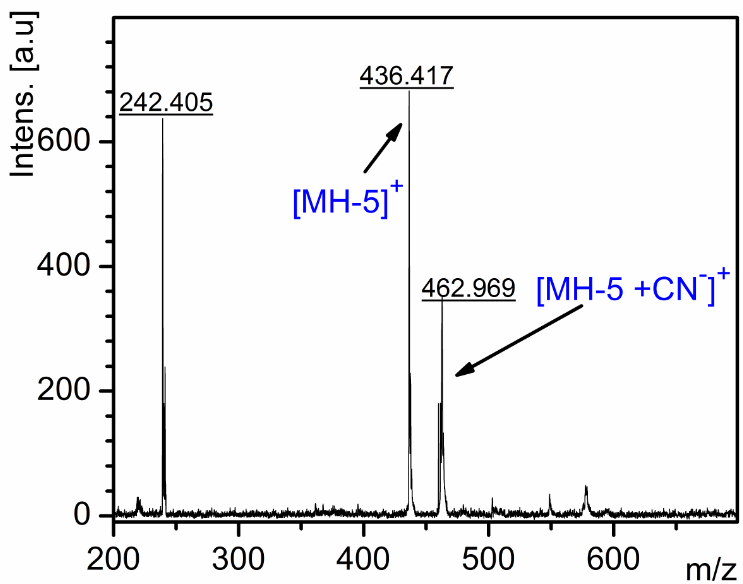


**Fig. S16.** MALDI-TOF spectrum of MH-5+CN^-^


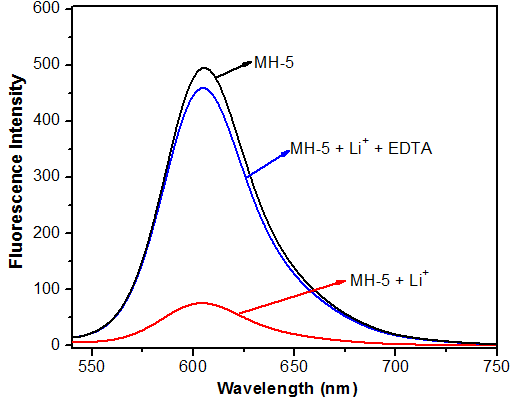


**Fig. S17.** The fluorescence spectra for the change in intensity of MH-5 in the presence of Li^+^ (1 equiv.) and EDTA (5 equiv.) DMSO-PBS buffer (10 mM, pH=7.25, v/v, 1:9).


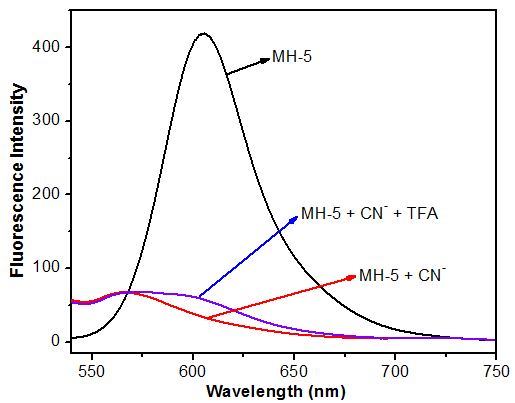


**Fig. S18.** The fluorescence spectra for the change in intensity of MH-5 in the presence of CN^-^ (1 equiv.) and TFA (5 equiv.) DMSO-PBS buffer (10 mM, pH=7.25, v/v, 1:9).


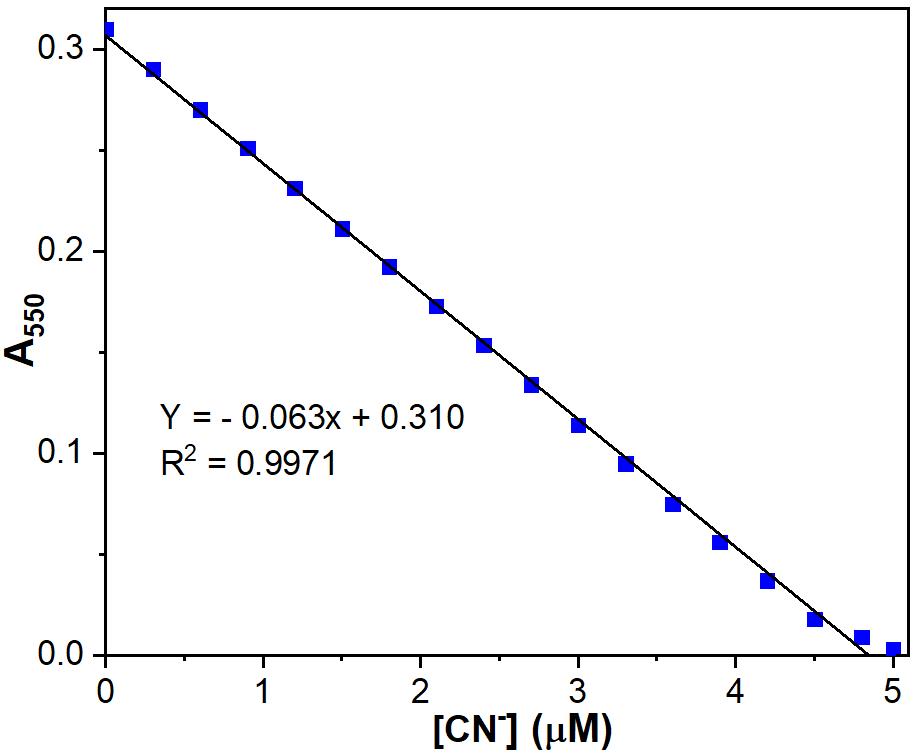


**Fig. S19.** Linear relationship between absorbance intensity and CN^-^ concentration (0.0-5.0 µM). Detection limit of MH-5 for CN^-^ by UV-Vis method (Measurements were taken in DMSO-PBS buffer (10 mM, pH=7.25, v/v, 1:9). [MH-5] = 5.0 µM)


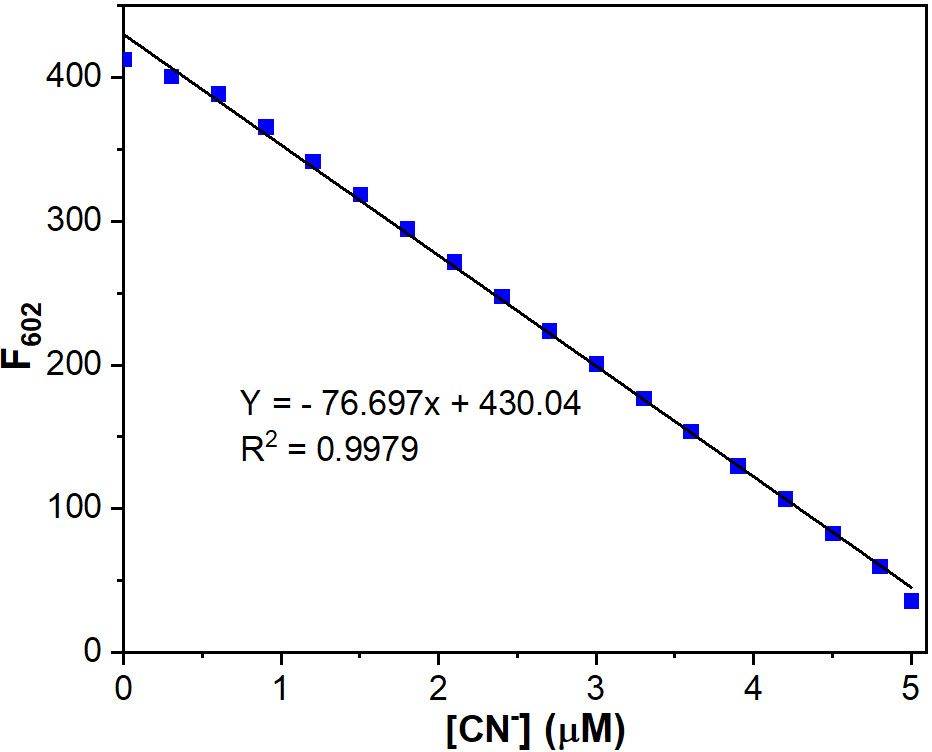


**Fig. S20.** Linear relationship between fluorescence intensity and CN^-^ concentration (0.0-5.0 µM). Detection limit of MH-5 for CN^-^ by fluorescence method (Measurements were taken in DMSO-PBS buffer (10 mM, pH=7.25, v/v, 1:9). [MH-5] = 5.0 µM)


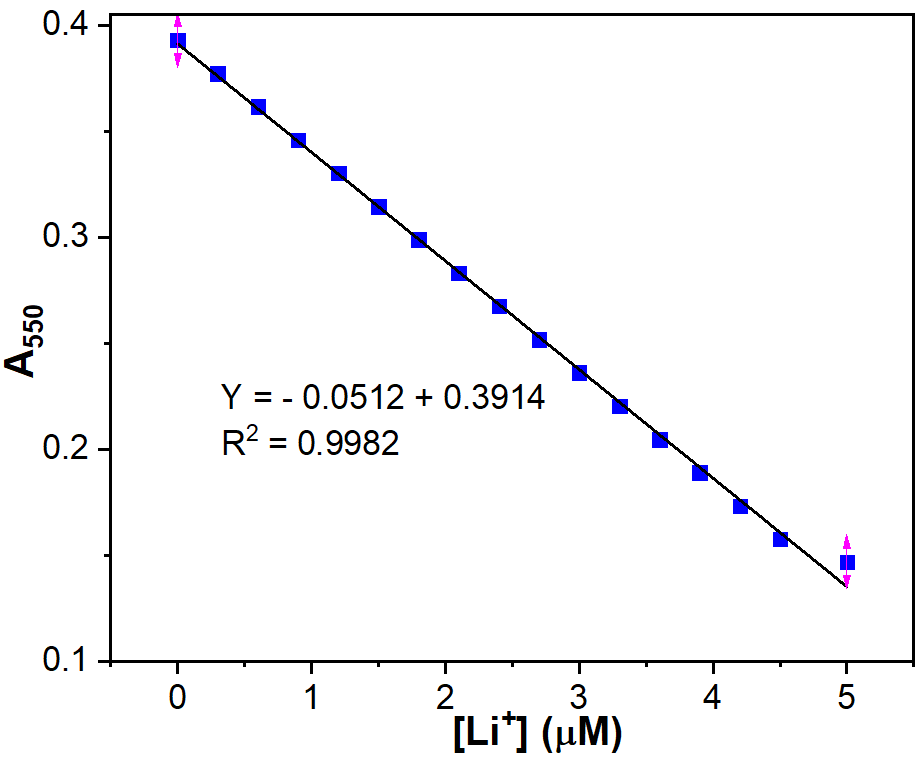


**Fig. S21.** Linear relationship between absorbance intensity and Li^+^ concentration (0.0-5.0 µM). Detection limit of MH-5 for Li^+^ by UV-Vis method (Measurements were taken in DMSO-PBS buffer (10 mM, pH=7.25, v/v, 1:9). [MH-5] = 5.0 µM)


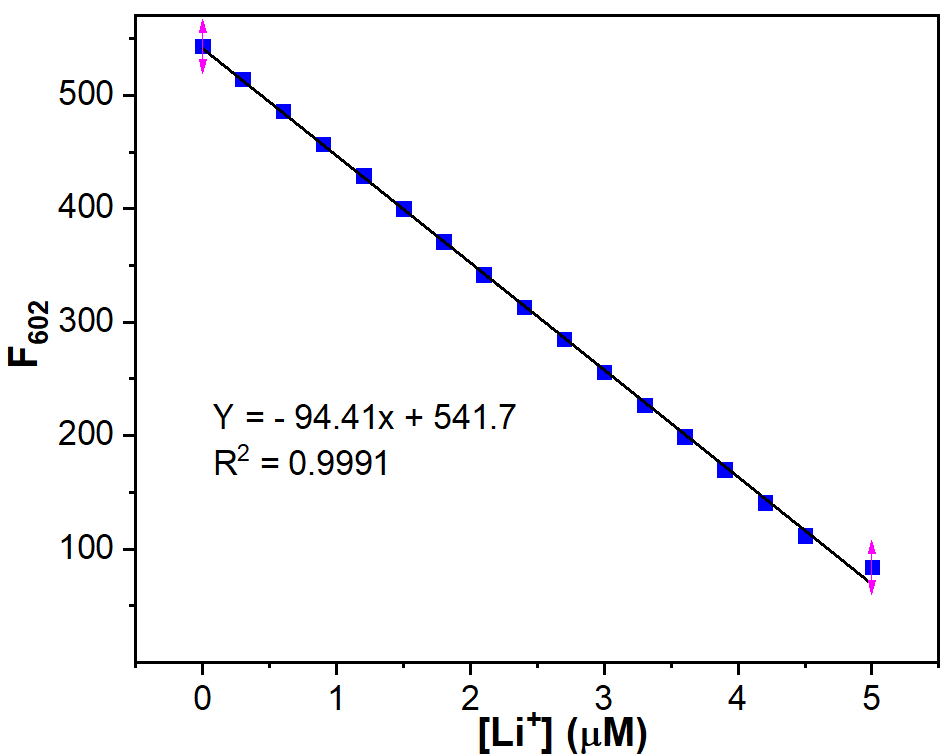


**Fig. S22.** Linear relationship between fluorescence intensity and Li^+^ concentration (0.0-5.0 µM). Detection limit of MH-5 for Li^+^ by fluorescence method (Measurements were taken in DMSO-PBS buffer (10 mM, pH=7.25, v/v, 1:9). [MH-5] = 5.0 µM)

**Table S1.**Comparison of several reported sensors for the detection of CN- and Li+

| **Sensor Structure** | **Testing Media (v/v)** | **Detected Anions or**  **Metals** | **Response Time** | **Detection limit (µM)** | **Application** | **Ref.** |
| --- | --- | --- | --- | --- | --- | --- |
|  | H_2_O/THF  (1:9, v/v) | CN^-^ | NA | 0.62 µM | Paper strips | (1) |
|  | H_2_O/THF  (1:9, v/v) | CN^-^ | NA | 0.97 nM | Paper strips | (2) |
|  | ACN | CN^-^ | < 2 min. | 0.7 µM | Paper strips | (3) |
|  | ACN/HEPES Buffer  (7:3, v/v) | F^-^, HSO_4_^-^, CN^-^ | 15 min. | 700 µM | Bio-imaging | (4) |
|  | DMSO/H_2_O  (1:2, v/v) | CN^-^ and Hg^2+^ | NA | 2.23 µM | Paper strips | (5) |
|  | DMSO/ PBS buffer  (1:9, v/v) | CN^-^ | 5 min. | 0.154 µM | Bio-imaging | Present Study  (for CN^-^) |
| 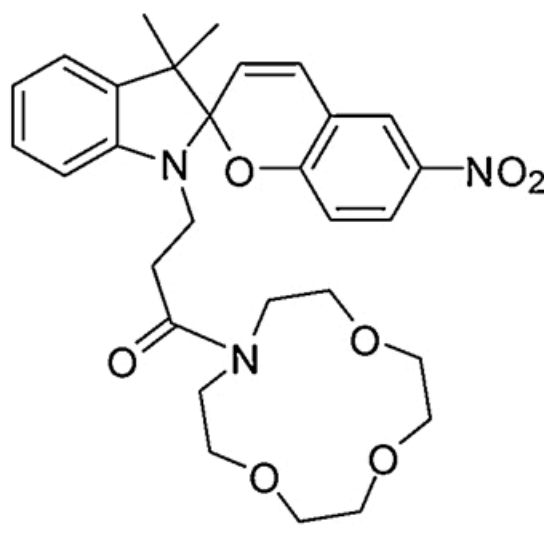 | ACN/PBS  Buffer  (1:1, v/v) | Li^+^ | 30 min. | 4.67µM | Bio-imaging | (6) |
|  | ACN/H_2_O  (3:7, v/v) | Li^+^ | NA | 0.37 µM | None | (7) |
| 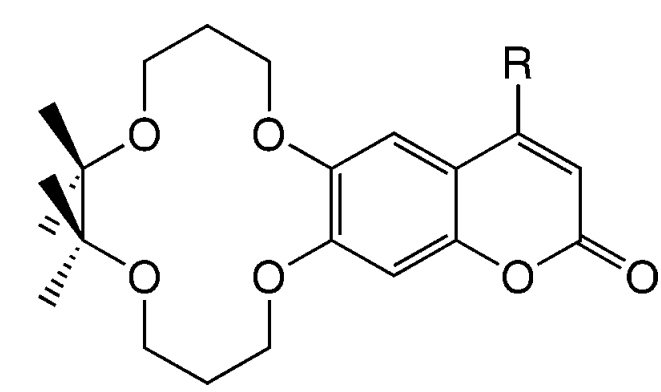 | MeOH/H_2_O  (1:99, v/v) | Li^+^ | < 2 min. | 600 µM | clinical  samples | (8) |
| 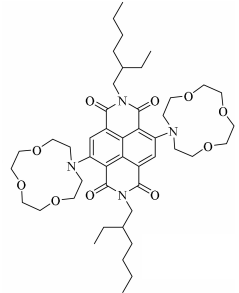 | ACN | Li^+^ | NA | 5.0 µM | None | (9) |
|  | ACN | Li^+^ | NA | 21 µM | None | (10) |
|  | DMSO/ PBS buffer  (1:9, v/v) | Li^+^ | 2 min. | 0.150 µM | Cell imaging | Present Study  (for Li+) |

NA: not available

**References**

1. Padghan SD, Hu J, Wang L, Wang F, Chen K. Low‐Molecular‐Weight J‐Aggregate Solid Red Emitter for Selective and Quantitative Detection of Cyanide. Chemistry An Asian Journal. 2023 Apr 3;18(7):e202201293.

2. Manivannan R, Son YA. Blue light emitting fluorophore for the effective detection of cyanide ion and electronic test kit development for real time measurement. Dyes and Pigments. 2023 Feb;210:110941.

3. Lyngdoh Lyngkhoi D, Khatua S. A coumarin containing hemicyanine-based probe for dual channel detection of cyanide ion. Inorganica Chimica Acta. 2024 Nov;572:122300.

4. Bej S, Das R, Mondal A, Saha R, Sarkar K, Banerjee P. Knoevenagel condensation triggered synthesis of dual-channel oxene based chemosensor: Discriminative spectrophotometric recognition of F−, CN– and HSO4− with breast cancer cell imaging, real sample analysis and molecular keypad lock applications. Spectrochimica Acta Part A: Molecular and Biomolecular Spectroscopy. 2022 May;273:120989.

5. Hosseinjani-Pirdehi H, Mahmoodi NO, Pasandideh Nadamani M, Taheri A. Novel synthesized azo-benzylidene-thiourea as dual naked-eye chemosensor for selective detection of Hg2+ and CN¯ ions. Journal of Photochemistry and Photobiology A: Chemistry. 2020 Mar;391:112365.

6. Kang J, Li E, Cui L, Shao Q, Yin C, Cheng F. Lithium ion specific fluorescent reversible extraction-release based on spiropyran isomerization combining crown ether coordination and its bioimaging. Sensors and Actuators B: Chemical. 2021 Jan;327:128941.

7. Kumari S, Joshi S, Sarmah A, Pant D, Sakhuja R. Highly Selective Sensing of Li+ in H2O/CH3CN via Fluorescence ‘Turn-on’ Response of a Coumarin-Indole Linked Dyad: an Experimental and Theoretical Study. J Fluoresc. 2016 Nov;26(6):2177–85.

8. Citterio D, Takeda J, Kosugi M, Hisamoto H, Sasaki S ichi, Komatsu H, et al. pH-Independent Fluorescent Chemosensor for Highly Selective Lithium Ion Sensing. Anal Chem. 2007 Feb 1;79(3):1237–42.

9. Hangarge RV, La DD, Boguslavsky M, Jones LA, Kim YS, Bhosale SV. An Aza‐12‐crown‐4 Ether‐Substituted Naphthalene Diimide Chemosensor for the Detection of Lithium Ion. ChemistrySelect. 2017 Dec 11;2(35):11487–91.

10. Kim H, Koo B. Lithium sensors based on photophysical changes of 1-aza-12-crown-4 naphthalene derivatives synthesized *via* Buchwald–Hartwig amination. RSC Adv. 2022;12(49):31976–84.
